# Pathophysiology in cortico-amygdala circuits and excessive aversion processing: the role of oligodendrocytes and myelination

**DOI:** 10.1093/braincomms/fcae140

**Published:** 2024-04-18

**Authors:** Giulia Poggi, Federica Klaus, Christopher R Pryce

**Affiliations:** Preclinical Laboratory for Translational Research into Affective Disorders, Department of Psychiatry, Psychotherapy and Psychosomatics, Psychiatric Hospital, University of Zurich, CH-8008 Zurich, Switzerland; Department of Psychiatry, University of California San Diego, San Diego, CA 92093, USA; Desert-Pacific Mental Illness Research Education and Clinical Center, VA San Diego Healthcare System, San Diego, CA 92093, USA; Preclinical Laboratory for Translational Research into Affective Disorders, Department of Psychiatry, Psychotherapy and Psychosomatics, Psychiatric Hospital, University of Zurich, CH-8008 Zurich, Switzerland; Neuroscience Center Zurich, University of Zurich and ETH Zurich, 8057 Zurich, Switzerland; URPP Adaptive Brain Circuits in Development and Learning (AdaBD), University of Zurich, 8057 Zurich, Switzerland

**Keywords:** cortico-amygdala circuit, myelin, oligodendrocyte, excessive aversion processing, psychiatric diseases

## Abstract

Stress-related psychiatric illnesses, such as major depressive disorder, anxiety and post-traumatic stress disorder, present with alterations in emotional processing, including excessive processing of negative/aversive stimuli and events. The bidirectional human/primate brain circuit comprising anterior cingulate cortex and amygdala is of fundamental importance in processing emotional stimuli, and in rodents the medial prefrontal cortex-amygdala circuit is to some extent analogous in structure and function. Here, we assess the comparative evidence for: (i) Anterior cingulate/medial prefrontal cortex<->amygdala bidirectional neural circuits as major contributors to aversive stimulus processing; (ii) Structural and functional changes in anterior cingulate cortex<->amygdala circuit associated with excessive aversion processing in stress-related neuropsychiatric disorders, and in medial prefrontal cortex<->amygdala circuit in rodent models of chronic stress-induced increased aversion reactivity; and (iii) Altered status of oligodendrocytes and their oligodendrocyte lineage cells and myelination in anterior cingulate/medial prefrontal cortex<->amygdala circuits in stress-related neuropsychiatric disorders and stress models. The comparative evidence from humans and rodents is that their respective anterior cingulate/medial prefrontal cortex<->amygdala circuits are integral to adaptive aversion processing. However, at the sub-regional level, the anterior cingulate/medial prefrontal cortex structure-function analogy is incomplete, and differences as well as similarities need to be taken into account. Structure-function imaging studies demonstrate that these neural circuits are altered in both human stress-related neuropsychiatric disorders and rodent models of stress-induced increased aversion processing. In both cases, the changes include altered white matter integrity, albeit the current evidence indicates that this is decreased in humans and increased in rodent models. At the cellular-molecular level, in both humans and rodents, the current evidence is that stress disorders do present with changes in oligodendrocyte lineage, oligodendrocytes and/or myelin in these neural circuits, but these changes are often discordant between and even within species. Nonetheless, by integrating the current comparative evidence, this review provides a timely insight into this field and should function to inform future studies—human, monkey and rodent—to ascertain whether or not the oligodendrocyte lineage and myelination are causally involved in the pathophysiology of stress-related neuropsychiatric disorders.

## Teaser

Poggi *et al*. provide a comprehensive comparative review of the current human and rodent evidence for associations and causal links between stress, oligodendrocyte/myelin alterations, cortico-amygdala circuit changes in structure-function and the excessive aversion processing that is typical of psychiatric illnesses such as depression, anxiety disorders and post-traumatic stress disorder.

## Graphical Abstract

**Graphical abstract:**
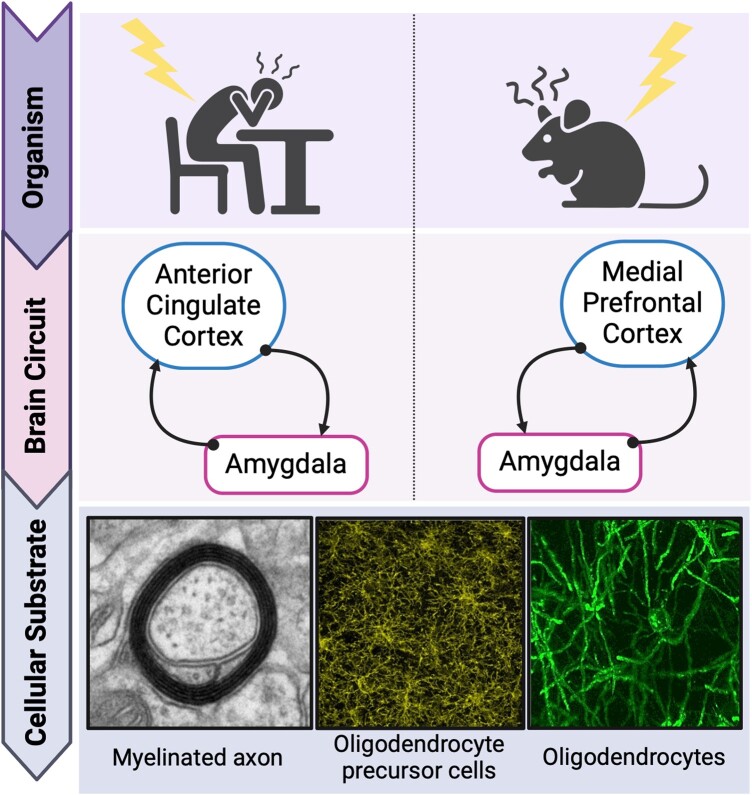

## The importance of the bidirectional cortico-amygdala long-range circuit in mediating negative valence processing and its psychopathology

Communication within the nervous system is based on the exchange of electrical information. Myelin, a proteolipid multi-layered insulating membrane, ensheaths the axons of neurons along almost their entire length, to achieve finely timed propagation of electrical impulses. Specifically, myelin confines the action potentials to the unmyelinated nodes of Ranvier and thereby enables fast saltatory conduction.^1-3^ In the central nervous system (CNS), myelin is generated by oligodendrocytes (OLs). The process of myelination begins after completion of formation of neuronal connections during the late embryonic stage and continues postnatally.^4-7^ The internodal distribution pattern of myelin varies substantially between grey and white matter, brain regions, axon subtypes, developmental stages and, potentially, axonal metabolic demands.^3,8-10^ Sub-cortical areas are more densely myelinated than cortical areas, which are more sparsely and lightly myelinated; in the human neocortex, including somatosensory, motor and visual regions, the process of myelination continues throughout adulthood.^11,12^

OLs are derived from oligodendrocyte precursor cells (OPCs).^4,13^ Part of the OPC pool reaches mature OL status already at early developmental stages.^14,15^ Other OPCs remain at the precursor stage and reside in the CNS into and throughout adulthood, as a potential reservoir of undifferentiated cells. These resident OPCs can differentiate and mature at later stages, including to support adult myelination.^14,16^ While there is substantial evidence for pre-programmed intrinsic development of OPCs,^14,16^ it is also the case that they can respond to a broad variety of external cues across the life span. Importantly, OPCs receive direct synaptic inputs from glutamatergic and GABAergic neurons, and can proliferate, migrate and differentiate in response to environmentally induced neural activity.^17-20^

In the present context, of particular relevance are the long-range axons of glutamatergic projection neurons, which form connections between brain regions that are spatially distant. Such connections are energy-demanding and completely dependent on myelination.^21-24^ Myelination along these fibres is essential for metabolic support of the axons, efficient action potential propagation, and resultant synchronization and connectivity between distant brain regions.^21-23,25^ The present review is concerned with two specific brain regions and the long-range myelinated axons that connect them bidirectionally. This circuit is of high relevance to the processing of emotional stimuli, including aversive stimuli, and is also implicated in stress-related psychiatric illnesses which present with pathologies of the so-called negative valence system.^26-29^ In humans and non-human primates, this is the bidirectional connection between the anterior cingulate cortex (ACC)—in particular, the rostral portion of the ACC including pregenual and subgenual subregions—and the amygdala.^30,31^ In rodents, specifically mouse and rat, this is the medial prefrontal cortex (mPFC), comprising the prelimbic (PrL) and infralimbic (IL) cortices and demonstrating some analogy with the primate ACC, including the bidirectional connection with the amygdala.^32^ In humans, this pathway contributes to the circuitry of emotion regulation.^33,34^ The amygdala is a major region for the processing of unconditioned and conditioned stimuli (CS) of emotional salience—both aversive and rewarding—and for the instigation of corresponding and integrated emotional responses. The human ACC contributes to the modulation of amygdala re-/activity, as well as receiving inputs from the amygdala.^31,33^ In rodents, evidence, in particular, that obtained using the paradigm of Pavlovian aversion learning and memory (PALM), indicates analogous roles for the mPFC and amygdala.^35-38^

Stressful or adverse life events, especially those of a chronic nature, impact the regulation of cognitive-emotional processing and are common risk factors for several neuropsychiatric disorders, including major depressive disorder (MDD), anxiety disorders (ADs) and post-traumatic stress disorder (PTSD).^39-44^ Clinical evidence suggests that behavioural changes are underlain by chronic stress-induced alteration of cortical regions including ACC: this involves, for instance, hypoactivation of ventral pgACC and hyperactivation of subgenual ACC. Stress also induces hyperactivation of amygdala.^26,44-49^ Rodent studies demonstrate causal evidence for inter-relationships between chronic stress, changes in mPFC and/or amygdala activity, and altered behaviour in tests of emotional stimulus processing.^50-55^ This comparative evidence raises the question of what is the pathophysiological process(es) via which chronic stress changes ACC-amygdala/mPFC-amygdala functioning? One promising candidate, based on their biological properties and the increasing evidence for their altered status in stress-related mental illnesses, is OLs and myelination within ACC/mPFC and amygdala, and along the long-range axons connecting them.^47,56-60^ Changes in the OL lineage status and subsequent myelin rearrangement could alter action potential propagation and ACC-amygdala/mPFC-amygdala synchronization.^25^ Regional heterogeneity in OL lineage status and myelination may contribute to region-specific disorder development.^61-65^ This could involve dysregulation in physiological cortico-amygdala circuit functioning and consequent dysfunctioning in emotional processing.^66^

## Roles of anterior cingulate cortex/medial prefrontal cortex<->amygdala circuitry in adaptive aversion processing

### Connections between anterior cingulate cortex and amygdala involved in aversion processing and behaviour in humans

**Figure 1:**
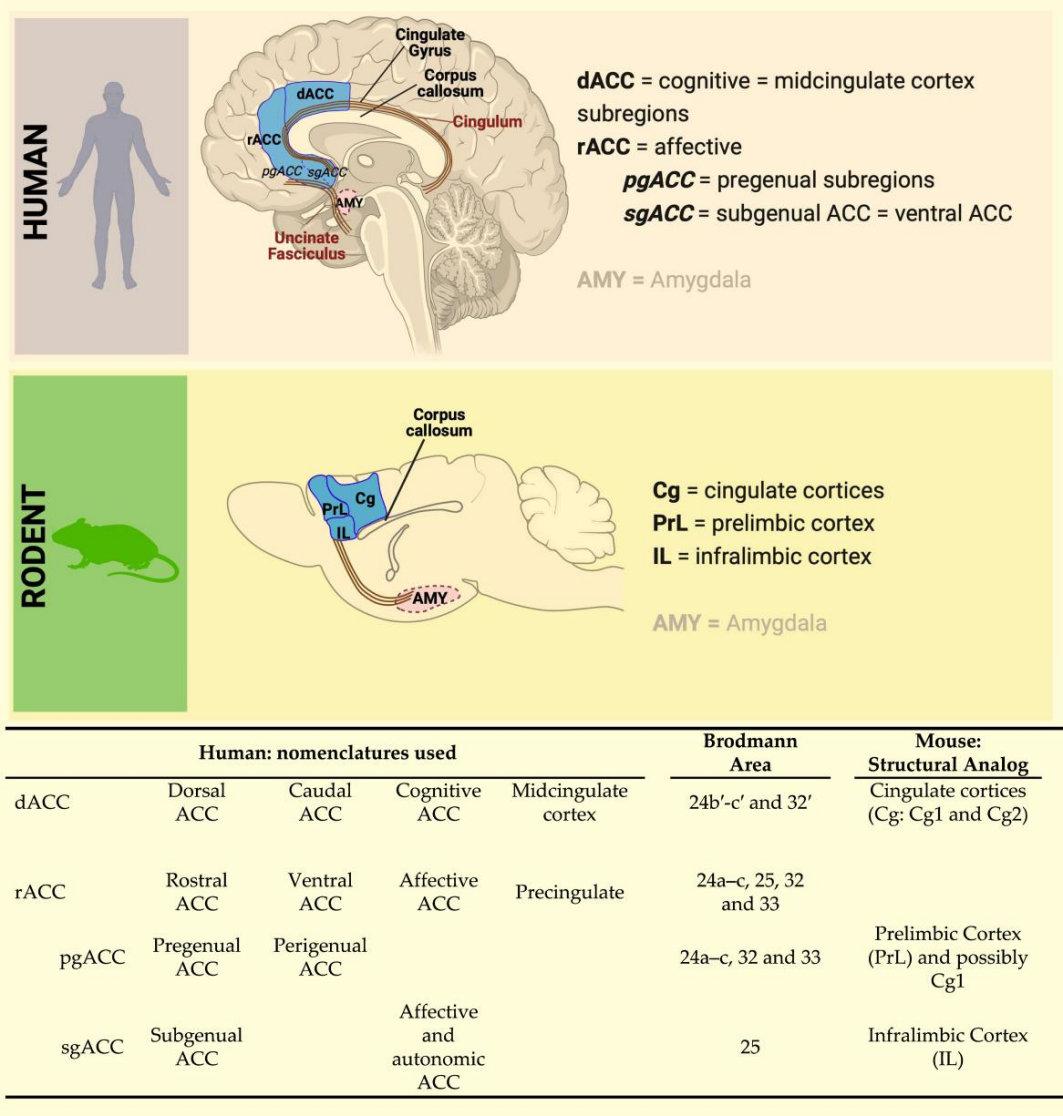
**Comparative anatomy of human anterior cingulate cortex and rodent medial prefrontal cortex.** The dACC is considered the cognitive area of the cingulate cortex and shows cytoarchitectural analogies with the cingulate Cortices 1 and 2 in rodents. The rostral anterior cingulate cortex, including the perigenual (rACC-pgACC) and the subgenual (rACC-sgACC) anterior cingulate cortex, is considered the affective and autonomic region of the anterior cingulate cortex and shows cytoarchitectural and connectivity analogies with the PrL and IL cortices in rodents (see also^71-73^). The illustration was created with Biorender (https://www.biorender.com).

The human prefrontal cortex (PFC) is a relatively large and heterogeneous brain region.^67^ It comprises two evolutionarily distinct tissue types with respect to granule cells, namely agranular and granular (including dysgranular). Granular cortex appeared later in evolution and is characteristic of tissue in extant primates; agranular cortex evolved earlier and is common to all extant mammals including rodents.^68^ The ACC of humans and other simian primates (apes and monkeys) is cytoarchitectonically agranular. It can be subdivided into dorsal ACC (dorsal anterior cingulate cortex (dACC), Brodmann's area (BA) 24) and rostral ACC (rACC, also partly BA24, BA25, BA32). The dACC, also referred to as the cognitive ACC, is connected with the PFC, parietal cortex and motor cortex. The rACC, also referred to as the affective ACC, includes pregenual ACC (pgACC, BA 24/32) located rostrally to the genu of the corpus callosum, and subgenual ACC (sgACC, BA 25) located ventrally to the genu of the corpus callosum (Fig. 1). The pgACC innervates all layers of the sgACC and, via these dense projections, regulates sgACC activity.^69^ The rACC is connected with the anterior insula, hippocampus, hypothalamus, nucleus accumbens and, of particular importance here, the amygdala.^70^

The amygdala is an almond-shaped structure located in the temporal lobe and, based on its uniformity across living species, has remained rather conserved during mammalian evolution. It comprises nuclei that are extensively inter-connected and differ from each other with respect to cytoarchitectonic and connectivity properties.^74-76^ It is broadly accepted that the amygdala can be subdivided into two divisions with respect to evolutionary emergence: (i) a primitive cortico-medial region, including cortical, medial and central nuclei; and (ii) a more recent basolateral region, including lateral, basolateral and accessory basal nuclei.^75^ The lateral nucleus is considered the main entry point of sensory stimuli, and the central nucleus the main output station^75^; these two are connected via the intermediate glutamatergic projections of the basolateral nucleus (BLA, also referred to as the basal nucleus).^74,75^

The medial temporal lobe (MTL), including amygdala and hippocampus, is connected to the PFC via white matter association tracts, including the uncinate fasciculus and cingulum. The uncinate fasciculus is the most medial of these and connects the sgACC to MTL including the amygdala.^77,78^ The cingulum is another of the tracts and connects dACC and sgACC with MTL, again including the amygdala.^71^ Indeed, these two tracts include the entire bidirectional axonal connections between ACC and amygdala.^79^ For primates, there are descriptions of high-density projections from sgACC to amygdala and of moderate density projections from pgACC to amygdala.^38,68,80,81^ In the opposite direction, dense projections originating in basolateral or accessory basal amygdala terminate in layers II–III and V of pgACC and sgACC.^82^ For caudal-medial BA24, medial BA25 and BA32, there are more ACC projections to the amygdala than are incoming from the amygdala.^81^ Such connectivity suggests a significant involvement of sgACC and pgACC in the regulation of amygdala activity and function.^38^

Regarding the functions of the human/primate ACC-amygdala circuit, there is substantial evidence that it is important in the regulation of emotional processing/responding, including to aversive stimuli. The amygdala and rACC are already activated during subliminal (implicit) emotional processing, whereas the dACC is particularly engaged during supraliminal (explicit) information processing.^83^ Electrical stimulation of the ACC has been reported to lead variously to fear, pleasure or agitation.^84^ Stimulation of pgACC specifically, as conducted in an epileptic patient who underwent stereotactic cingulotomy, led to an increase in self-report of fear feelings.^70,79^ Further direct evidence is provided by case studies with brain-lesion patients: lesions of the ACC are related to a wide range of changes in emotional stimulus responding and concomitant behaviour, including emotional blunting, apathy, impaired facial expression identification (especially fear), lower threshold for fear or startle responding, changes in aggressivity, deficits in theory of mind and in conflict adaptation in emotional tasks, and reduced responding to painful stimuli.^70,84,85^ Lesions of the amygdala are related to reduced anxiety in humans^86^ and reduced attention to threatening events/stimuli in macaques.^87^ Therefore, human and monkey lesion-study data indicate that rACC and amygdala are involved in the regulation of emotional processing.

Imaging studies in healthy humans provide functional evidence for the involvement of ACC-amygdala connectivity in responding to negative stimuli. Firstly, volumetric analysis showed that low rACC thickness was associated with high amygdala volume in individuals with relatively high negative affect scores.^88^ Expectation of an unpleasant stimulus increased functional magnetic resonance imaging (fMRI) activity (i.e. bold-oxygen-level-dependent (BOLD) signal) in both BA32 and amygdala.^89^ In another fMRI study in healthy subjects, partial-reinforcement fear conditioning was conducted, which leads to delayed extinction learning. An rACC subregion, referred to in the paper as sgACC but mapped as slightly anterior to BA25,^90-92^ was engaged throughout. The direction of change in BOLD signal relative to resting state depended on the trial type: sgACC activity was decreased during acquisition learning trials and increased during extinction learning trials; somewhat in contrast, amygdala activity was increased during acquisition learning trials and decreased during the first day of extinction learning trials. Furthermore, a lower depression of this cortical subregion activity correlated with a higher degree of extinction learning and with reduced amygdala activity during the early stage of the extinction retention phase.^92^ In a colour flanker task-fMRI study, healthy participants were presented with either emotionally negative or neutral coloured words, and were asked to determine the ink colour of a centrally presented word which was flanked by the emotional/neutral word, either in the same (congruent) or in a different (incongruent, i.e. conflicting) colour.^93^ In trials where emotionally negative words were presented, bilateral amygdala activation occurred.^93^ Furthermore, in incongruent trials, i.e. trials with conflict, the rACC was activated and conflict processing was more rapid. Increased functional connectivity (FC) between rACC-amygdala (and rACC-dACC) occurred in incongruent-emotional trials, i.e. trials with a conflict and a negative emotional stimulus,^93^ suggesting that rACC integrates conflict and emotional information, while receiving input from the amygdala.^93^ In a real-time fMRI (rt-fMRI) and neurofeedback study, healthy women were presented with pictures showing negative affective scenes and they were given feedback on their amygdala activation via a visually presented thermometer. They were instructed to attempt to either downregulate or upregulate the activity of their amygdala, or to look at pictures without any feedback. The study revealed that active regulation of amygdala activity engaged the rACC; specifically, increased amygdala response to emotional stimuli was associated with reduced sgACC activity, and reduced amygdala response to emotional stimuli was associated with increased activation of sgACC.^94^ In another study employing rt-fMRI and neurofeedback training, healthy volunteers were presented with affectively negative and neutral pictures and their amygdala activity was measured. They were instructed to use cognitive reappraisal to regulate their emotional response and underlying amygdala activity.^95^ Such neurofeedback training of emotional regulation via inhibition of amygdala activity led to increased rACC (BA32)-amygdala FC.^95^

Partially in contrast with the above findings, increased activity in the sgACC associated with higher negative affect,^49,96^ and activation of pgACC/BA32 (but not sgACC/BA25) in fear extinction recall,^97^ have also been observed in healthy subjects. Specifically, in a fMRI study, healthy subjects were instructed to recall aversive autobiographical memories while using cognitive strategies to ruminate on the memories (feel strategy), or to actively prevent rumination using methods derived from cognitive behavioural therapy (accept or analyse strategies).^96^ The sgACC was mostly active during the ‘feel strategy’, and minimally involved in the application of the other two (coping) strategies.^96^ Furthermore, in an event-related fMRI study, healthy subjects underwent a 2-day fear conditioning and extinction protocol. Briefly, on Day 1 (conditioning phase), the subjects were exposed to two different CS paired to an aversive unconditioned stimulus (US), and only one of the two CSs was extinguished on Day 1 (extinction phase); on Day 2, both CSs were presented in the extinction context of Day 1 (extinction recall). During the conditioning phase, increased amygdala activity and decreased pgACC and sgACC activity were detected; during the late extinction learning phase, increased activity of amygdala, pgACC and sgACC was observed, while at extinction recall on Day 2, sgACC was not further engaged.^97^

The relationship between rACC and amygdala in the processing of aversive stimuli is complex and certainly not one of simple mutual inhibition. Indeed, the between-region FC evidence for the rACC-amygdala circuit suggests that finely tuned bidirectional regulation of activity involving rACC and amygdala underlies situation-appropriate emotional processing and decision-making, and subsequent behavioural outcome. This is likely due to specific anatomical subregions mediating the various inhibitory and excitatory effects observed, and caution is warranted in the interpretation and comparison of findings.^49^

### Connections between medial prefrontal cortex and amygdala involved in aversion processing and behaviour in rodents

While the amygdala is considered to be relatively evolutionarily conserved across extant mammalian orders including rodents and primates, cortical regions have undergone a more substantial enlargement and specialisation, and this is certainly the case in simian primates compared with rodents.^35,82,91,98^ In rodents, the cortex is entirely agranular^68^; consequently, when comparing rodent and primate cortices and respective functions, the focus must be on the agranular regions in both groups.^99,100^ With regards to the region of the rodent cortex that has analogy to the primate, including human, ACC, there is not a definitive consensus. Anatomical, cytoarchitectural and connectivity studies provide substantial evidence that the mPFC, comprising PrL and IL cortex, is the primary candidate (Fig. 1). Important here is the principle that the spatial organization of brain regions relative to each other is highly conserved in evolution and therefore in extant species across mammalian orders.^68^ Applying this principle, it has been proposed that rodent IL and PrL are approximate analogues of primate sgACC (BA25) and pgACC (BA32, 24), respectively,^38,91,100,101^ and that rodent cingulate cortex is an approximate analogue of primate dACC (dorsal BA24)^38,101^ (Fig. 1). Lesion studies of rodent mPFC demonstrate a wide range of effects on emotional stimulus responsiveness and concomitant behaviour, including emotional blunting, disruption of mating behaviour, lower threshold for fear or startle responding, reduced aggressivity and reduced response to painful stimuli.^70,102^ Similar to the primate ACC subregions, the rodent IL and PrL are highly and reciprocally inter-connected^103^ as well as densely connected with sub-cortical regions involved in emotional processing.^100,104^ Although not commonly stated, a strong case can be made for rodent emotional stimulus processing being entirely implicit.^105^

Concerning their connectivity with the amygdala, both PrL and IL send and receive dense projections to and from the BLA, without interlaminar signalling.^32,103,106-110^ The glutamatergic mPFC-BLA and BLA-mPFC projections form synapses onto pyramidal neurons and/or GABA interneurons in the respective efferent region. The spontaneous firing activity of the majority of mPFC neurons is inhibited by BLA stimulation,^106,111,112^ and local circuit GABA interneurons fine-tune the glutamate neuron activity in these circuits.^36,113,114^ At the functional level, these anatomical features result in complex PrL-BLA/BLA-PrL and IL-BLA/BLA-IL interplay between glutamate-neuron to glutamate-neuron excitatory loops, including positive feedback, and between glutamate neuron to GABA-interneuron to glutamate neuron pathways, including feedforward inhibition. By means of the latter, the mPFC is regulated by and in turn regulates amygdala activity in response to emotionally relevant stimuli. Synchronization of the activity in bidirectional mPFC-BLA circuits would appear to be fundamental to adaptive regulation of emotional processing.^32,38,100,103,107,108,110,112,113,115,116^ Bidirectional projections between BLA and mPFC have been demonstrated to be involved in the regulation of responsiveness to aversive and rewarding stimuli, including social stimuli, as shown by manipulations of this circuit using optogenetic approaches^32,110,117-119^ and lesioning or pharmacological inactivation,^68,120^ as well as by correlational studies using cell firing measures,^121^ extracellular recording and local field potential (LFP) measures,^112,116^ whole-cell recording^32^ and circuit-specific c-Fos expression.^110,118,122,123^

The following summarizes relevant studies of the role of mPFC<->BLA circuits in regulating aversion-directed behaviour in unmanipulated (‘healthy’) mice. While a few studies have reported on the role of the mPFC<->BLA circuit in active avoidance behaviour (see e.g.^124,125^), most of the findings are based on Pavlovian ‘fear’ conditioning (or PALM), and on anxiety readouts in the elevated plus maze (EPM) test and the open field (OF) test. The PALM paradigm involves learning the predictive association between an initially neutral stimulus, usually a tone, and an innate aversive stimulus (US), usually a moderately intense and brief electric footshock. The tone becomes a CS to the US and elicits a behavioural conditioned response of freezing during learning and subsequent memory recall; more time spent freezing equates to a higher level of aversion processing. The EPM test involves placing the rodent on an elevated plus-shaped frame comprising two arms with walls (‘closed arms’) and two arms without walls (‘open arms’). This presents a choice, or conflict, between either remaining in the relatively sheltered closed arms or exploring the relatively exposed (aversive) open arms; the neurobehavioural state induced by resolving this conflict constitutes anxiety, and more time spent in the closed arms equates to a higher level of anxiety. The OF test involves placing the rodent in an arena comprising relatively sheltered walls/corners and a relatively exposed (aversive) centre; as for the EPM, this presents a choice and the neurobehavioural state induced by resolving the conflict of whether or not to enter the centre constitutes anxiety.

With respect to the BLA-to-mPFC pathways, these are essential to retain information about aversion associations.^110,119^ For example, in mice, recording LFPs during aversion memory expression or OF testing showed that aversion exposure increases theta power in the BLA and theta synchrony between BLA and mPFC.^116^ Direct optogenetic stimulation of BLA-to-mPFC pathways induces anxiogenic effects, whereas inhibition leads to anxiolytic effects, measured in OF and EPM.^117^ It is important to note that these studies did not differentiate between PrL and IL. BLA glutamatergic neurons projecting to PrL or IL are proposed to be activated by different contingencies, namely aversion learning, consolidation, expression or extinction.^32,98,110^ Specifically, BLA-to-PrL neurons fired in response to cues conditioned to electric footshock, and stimulation of this pathway led to increased aversion memory expression.^121^ Furthermore, aversion learning was associated with increased c-Fos expression in BLA-to-PrL neurons. By contrast, aversion extinction learning was associated with increased c-Fos expression in BLA-to-IL projection neurons; consistent with causality in this association, inhibition of BLA-IL projecting neurons impaired aversion extinction learning.^110^ The processing of aversive information in the BLA-to-mPFC pathways during learning, memory expression and extinction might also lead to bidirectional firing of neurons between PrL and IL.^119^

With respect to mPFC-to-BLA, also these projections are essential for adaptive and flexible processing of aversive versus neutral stimuli.^116^ In learned-innate (i.e. PALM) and innate (i.e. OF) protocols, stimulus discrimination and safety detection are associated with increased theta-frequency synchrony between mPFC and BLA, with relative theta power being greater in the mPFC-to-BLA than the BLA-to-mPFC pathway.^116^ This is consistent with a role for mPFC in the regulation of BLA activity.^116^ The PrL and its PrL-to-BLA projections are recruited during aversive memory expression and renewal, but not during retrieval of extinction memory. The IL and its IL-to-BLA projections are not required for aversive memory expression but are recruited for retrieval of extinction memory and fear suppression specifically.^112,120-123^

In summary, the rodent mPFC<->BLA circuit is fundamental to adaptive aversion processing and responding. The PrL and IL subregions of mPFC are activated during aversion learning and extinction, respectively. The BLA is activated during aversion stimulus processing and learning. Bidirectional BLA<->PrL activity underlies aversion learning and memory expression, and bidirectional BLA<->IL activity underlies aversion extinction learning. That is, the evidence is for mutual regulation, with high BLA-PrL neuron activity during aversion escalation and high IL-BLA neuron activity during aversion de-escalation. In this regard, there is some functional analogy with the human rACC-amygdala circuit. However, sub-regional analysis at structural and functional levels reveals that there is a dissociation between primate rACC and rodent mPFC: while structurally pgACC-PrL and sgACC-IL can be regarded as respective analogues, functionally, it is rather pgACC-IL and sgACC-PrL that are respective analogues.^91,126^ It is also the case that granular regions of the primate PFC, rather than the agranular ACC, may well be responsible for some of the functions mediated in rodents by the mPFC; for example, BA10 could be relevant in this regard,^49,90,126^ including in terms of regulating pgACC/sgACC activity.^90,91^ That is, as a result of cortical expansion, some cognitive-emotional functions mediated by the mPFC in rodents are mediated by PFC rather than ACC regions in humans and other primates.^90^ Accepting the need for caution, the findings of detailed, cause-effect rodent studies of mPFC<->BLA-mediated emotional processing are nonetheless of translational relevance to furthering understanding of rACC-amygdala-related emotional functions (and dysfunctions) in humans.

## Myelin: an essential biological substrate for adaptive functioning in ACC<->amygdala and mPFC<->BLA circuits?

The human ACC<->amygdala and rodent mPFC<->BLA circuits respond rapidly and proportionately to emotional stimuli: the subregions comprising these circuits display balanced reciprocal engagement and activation/inhibition to regulate stimulus processing and emotional behaviour. The neurons comprising these circuits require high temporal precision in action potential propagation, plasticity and adaptivity. In humans, the long-range axons of the glutamate neurons connecting ACC and amygdala are highly myelinated and located within the cingulum bundle and the uncinate fasciculus, two of the main white matter tracts of the CNS.^127,128^ Comparably, in rodents, the long-range glutamatergic fibres connecting mPFC and amygdala, are also highly myelinated.^22,129^ In addition to white matter tracts, within ACC and rodent mPFC and amygdala and rodent BLA, grey matter neuronal myelination has been demonstrated via various histological and biochemical approaches (e.g.^130-132^). Interestingly, and quite differently from the typical white matter pattern, grey matter myelination appears heterogeneous and especially light and sparse in cortical regions involved in higher brain functions. Also, the more superficial the cortical layer, the lower the proportion of grey matter myelin coverage.^11^ Additionally, axons of cortical pyramidal neurons and GABA interneurons present with a peculiarly patchy internodal myelin distribution.^62,65,133^

For decades, brain plasticity has been regarded as the exclusive domain of neurons and their synapses, with OL-derived myelin conferred the role of ‘static axon insulator’. Recently, however, there is mounting evidence that the OL lineage and myelination are also important in circuit (mal)adaptation, plasticity and network synchronization.^14,25,134-140^ This could well also be the case for the OL lineage and myelin within the ACC<->amygdala/mPFC<->BLA circuits. A diffusion tensor imaging study reported that trait anxiety in healthy adult men was associated with higher fractional anisotropy in uncinate fasciculus, suggesting increased myelin content.^141^ Neurofilament light chain, a neurofilament protein and cytoskeletal component in long myelinated axons, can be used as a peripheral marker of neuroaxonal injury, and was increased in and associated with reduced cognitive performance in depressed patients.^142^ Such studies indicate that changes in myelin content could directly contribute to alteration of higher brain functions, emotional responses and learning processes in humans, potentially by regulating brain circuit synchronization.^143,144^ In rodents, aversion learning and aversion extinction learning both require oligodendrogenesis and new myelination in mPFC,^137,145^ and preventing oligodendrogenesis impairs consolidation of contextual aversive memory.^137,145^ Moreover, social experiences, such as social isolation and social reintegration after isolation, lead to altered myelin content in the mPFC.^132,146,147^ Although the specific mechanisms that mediate OL lineage and myelin plasticity in the context of cognitive and emotional processing have not been fully determined, it has, for instance, been reported that motor learning (and related brain circuit activity) impacts on OL lineage proliferation and differentiation and, consequently, on myelination.^134-136,148,149^ It has been suggested that myelination might be specifically guided towards certain axons (but not to others) via axonal vesicular release^150^ and action potentials.^151,152^ Together, such evidence indicates the involvement of the OL lineage, myelin and adaptive myelination in emotional processing, including in the ACC<->amygdala and mPFC<->BLA circuits.

## Aversion-processing pathology in human stress-related affective disorders and rodent models of stress-induced excessive aversion processing

In this section, we first summarize the evidence for stress-related changes in aversion processing in the psychiatric illnesses of MDD, ADs and PTSD, focusing on negative-valence psychopathologies that implicate the ACC<->amygdala circuits. We then summarize the evidence for some of the rodent models that have been developed to investigate the causal inter-relationships between stress and excessive aversion processing, focusing on negative-valence behavioural processes that are mediated by the mPFC<->amygdala circuits.

### Aversion processing psychopathologies in MDD, ADs and PTSD

*MDD* is characterized by at least two consecutive weeks of the core symptoms of depressed mood (feelings of sadness, emptiness, hopelessness) and/or anhedonia, with other symptoms including thought of death, suicidal ideation, and appetite and sleep disturbances.^153,154^

*ADs* are characterized by excessive fear and anxiety and related behavioural and physiological disturbances.^153^ Each specific AD is distinguished by the stimulus or event that leads excessive to fear or anxiety processing.^153^ Affected individuals also present with symptoms of autonomic arousal (e.g. palpitation, sweating) and restlessness, high fatigability, difficulty in concentrating, irritability, sleep disturbance and muscle tension.^155^

*PTSD* is a trauma-/stressor-related disorder that develops after experiencing at least one traumatic event/stressor.^153,156^ Symptoms include hyper-alertness, emotional numbing, re-experiencing (in dreams or thoughts) the traumatic events, difficulties in concentrating and sleeping and guilt over the whole traumatic experience; also, symptoms could appear immediately or many years after trauma.

One feature common to each of these stress/trauma-related psychiatric disorders is altered—specifically excessive—aversion processing. Furthermore, excessive aversion processing pertains to both the aetiopathogenesis and the psychopathology of disorder. With respect to aetiopathogenesis, aversive life experiences/events are major epidemiological risk factors.^42,154,157^ For MDD, at any given time in life, stressful life events are associated robustly with the onset or worsening of the disorder.^154,157,158^ The association is intensified in the case of severe stressors^157^ and if the individual presents with certain personality traits, e.g. high neuroticism.^41^ For ADs and PTSD, natural disasters and life-threatening situations are associated robustly with disorder onset.^159,160^ The number of adversities experienced also appears to be important: exposure to multiple severe traumatic events increases the likelihood and severity of PTSD.^160^ Indeed, it has been suggested that chronic exposure to uncontrollable and unpredictable stressful life events leads: in MDD, to the assessment of all life events as uncontrollable, resulting in a generalized state of uncontrollability or helplessness, and the onset of MDD^161,162^; in ADs, to increased aversion hyper-sensitivity and the onset of ADs.^163^

With respect to psychopathology, as summarized above, excessive aversion processing and its associated behavioural and physiological states constitute one of the major symptoms of MDD and the major symptoms of ADs and PTSD. There is a shift, or bias, in the significance attributed to aversive events such that the responses they elicit are more marked, the time during which they are processed is increased (rumination), and the assessment of whether they are controllable is decreased (helplessness). Whilst much of the evidence for this aversion-dominated neuropsychological state is based on patient interview, it is also possible to quantify the increased aversion processing of MDD, ADs and PTSD using neuropsychological tests, for example, PALM.^154,155,164-167^ Similarly, treatment outcomes are mostly defined by clinical assessment, but additional assessment using neuroimaging can give insights into mechanisms underlying treatment success. A well-established first line intervention for MDD, AD and PTSD is cognitive-behavioural therapy (CBT). In a meta-analysis of whole-brain results from task-based fMRI studies of subjects with these disorders, CBT was shown to alter functional brain response, specifically in the mPFC, extending to the dACC. The altered responses were associated with a positive CBT outcome, highlighting the relevance of these regions for emotional regulation and as a predictor of psychotherapy outcome.^168^ It has been proposed that: patients with MDD have reduced prefrontal control over limbic regions in the circuitry of emotional processing; impaired emotional regulation is linked to activity in the ventrolateral PFC in MDD, and responsiveness to psychotherapy requires a brain state with sufficiently adequate connectivity in mood-regulating systems. Such connectivity could be needed, so that engagement of these systems via psychotherapy can reduce negative emotional states. Indeed, better responding to CBT in terms of symptom reduction was observed in MDD patients who were closer to healthy controls in terms of reactivity to emotional stimuli in the prefrontal cortex and ACC.^169^

### Rodent models of stress-induced hyper-sensitivity to aversion

In rodents, in contrast to humans, it is possible to conduct controlled experiments in which the effects of specific stressors on brain and behaviour are investigated. Such preclinical models require specific validity, namely: (i) they must include a translational stressor as the triggering factor for negative valence system dysfunction (aetiological validity); (ii) they must include behavioural and/or brain-activity readouts relevant to those observed in the disorder (face validity); (iii) if known for the disorder, they should present with anatomical and biochemical alterations comparable to those observed in the disorder (construct validity). Here, we present some of the rodent models of stress-induced increased aversion processing, of relevance to MDD, ADs and/or PTSD.

One example of a mouse model relevant to the state of hyper-sensitivity to aversion that exists in adult-onset MDD and ADs is chronic social stress (CSS)-induced excessive PALM. In this model, CSS comprises 15 days of distal sensory exposure of an adult male mouse to an aggressive, dominant mouse together with a short daily proximal exposure including physical attack. The stressor is uncontrollable in that during proximal exposure the submissive behaviour of CSS mice does not prevent attack; it is also unpredictable in that the aggressor mouse is different on each day.^170,171^ Compared with control (CON) mice, CSS mice display generalized excessive PALM, measured as increased time spent in defensive freezing behaviour, a neurobehavioural state relevant to MDD and ADs.^170,172-174^ In addition to increased PALM to an inescapable footshock, CSS mice also display decreased escape behaviour to an escapable footshock; this is consistent with a deficit in controllability, a neurobehavioural state relevant to increased helplessness in MDD.^170,175^ To model some aversion hyper-sensitivity states that occur in PTSD, adult mice are exposed to a single high-intensity electric footshock without a CS. Relative to controls, such mice spend a high proportion of time freezing when exposed to the arena (context) in which they were shocked, relevant to associative aversion in PTSD. Furthermore, they also show a high freezing response when exposed to a neutral tone in a novel environment, relevant to non-associative (generalized) aversion hyper-sensitivity and hyper-arousal in PTSD.^176^

In addition to adulthood, rodents can be exposed to stressors during specific stages of development, and states of aversion hyper-sensitivity can then be measured in juveniles/adolescents or adults.^177^ For example, maternal separation combined with limited nesting/bedding material during postnatal Weeks 1–2 leads to increased anxiety-relevant behaviour in adult rats and mice, including reduced ultrasonic vocalization in contextual and cue PAL,^178^ increased freezing in PALM,^179,180^ altered locomotion in OF, and reduced open arm entries in EPM.^179,181^ Early weaning, alone or combined with juvenile chronic unpredictable stress (including forced swim, footshock, restraint, tail pinches, immobilization and cold exposure), leads in adulthood to increased anxiety in EPM and OF.^53,182^ In rats and mice, juvenile short-term variable (unpredictable) stress (including restraint, EPM, forced swim), juvenile social defeat and peripubertal stress (including OF, EPM and predator odour), lead in adulthood to reduced time spent in open arms of the EPM and the centre of the OF.^183-185^ As a last example, post-weaning social isolation leads to reduced activity in OF in male mice and rats^186-188^ but not in female mice,^186^ and to increased retention of aversive memory.^189^

Therefore, in rodents, combining stressors with behavioural readout tests can recapitulate the relationship between aversion aetiopathogenesis and excessive aversion processing psychopathology that pertains in human MDD, ADs and PTSD. Crucially, the animal model studies demonstrate that this relationship is causal. In the above, we have focused on those negative-valence psychological/behavioural states that involve the ACC/mPFC<->amygdala circuits as major hubs in their overall underlying neural circuitry. In the next two sections of this review, we assess the evidence for involvement of changes in OL and/or myelination status in these circuits, both in human stress-related psychiatric disorders with excessive aversion processing and in rodent models thereof. This is done firstly for in vivo studies and secondly for post-mortem/ex vivo studies.

## Evidence for and against myelin-related structure-function changes in anterior cingulate cortex/medial prefrontal cortex<-> amygdala circuitry underlying excessive aversion processing

### Human ACC<->amygdala myelin-related structure-function changes in stress-related psychiatric disorders

Structural MRI can be used to study brain volumetry and diffusion-based MRI to study the orientation and integrity of white matter tracts by measuring the water diffusion in neural tissue.^190,191^ A commonly used voxel-wise measure is fractional anisotropy (FA), which estimates the degree to which tissue organization limits diffusion of water molecules in brain white matter. FA is in part dependent on myelination of axons, such that decreased FA is a biomarker for diminished myelination integrity. Mean diffusivity (MD) is a measure of the overall diffusion within a voxel and increased MD is consistent with decreased myelination.^192^ Relevant to other microstructural changes in white matter, further measures include radial diffusivity (RD) (water diffusion perpendicular to the axonal wall) and normalized number of fibres (representing fibre geometry in white matter tracts).^193^ Functional MRI for the study of FC of BOLD signals in terms of their temporal correlations within and between spatially distributed brain regions includes (i) resting-state (rs-)MRI, investigating spontaneous temporal correlations, and (ii) task functional (f)MRI, investigating such temporal correlations during the performance of a task.^71^ For both rs-MRI and fMRI, it is assumed that regions with temporally correlated activities constitute functional networks.^194^

The ACC and amygdala, in addition to their bidirectional structural connections, are involved in basic FC networks. These include the salience network (SN), which is involved in the processing of stimulus salience^71^: The main region of the SN is the dACC, and it also includes the amygdala.^71^ Using seed-based approaches, a wide distribution of ACC connectivity has been identified^71,195^: in general, the rACC has relatively strong FC with dACC and medial prefrontal regions, and the sgACC with amygdala and hippocampus (see also section 2.1).^196^ In the following sections, we provide a summary of the evidence for involvement of structural-functional changes in ACC<->amygdala in one or more of MDD, ADs and PTSD. Clearly, FC measured using rs-MRI or task fMRI is dependent on myelination of the neuronal axons within and between brain regions. Equally clearly, however, differences in FC cannot be interpreted as evidence for differences in myelination. Strong FC is common between regions with no direct structural connection, cautioning against the inference of structural connectivity from FC.^197^ Given this and the aims of this review, we limit ourselves to presenting the findings of those papers that report both ACC>->amygdala MRI FC data and structural MRI data (Supplementary Table 1).

#### Findings in adolescent patients

The adaptive reorganization of both myelination and white matter volume that occurs between adolescence and adulthood^198^ makes adolescence a valid starting point to draw inferences about potential premorbid pathological changes in brain structure relevant to MDD, ADs and PTSD aetiopathophysiology. In adolescents, volumetric studies have demonstrated lower right-hemisphere whole volumes of ACC white and grey matter in MDD versus control participants.^199^ When investigating the sgACC specifically, no main group difference between adolescent MDD and control participants was found.^200^ Nevertheless, there was an inverse relationship between sgACC volume and depression scores within MDD participants, and smaller sgACC volume was associated with comorbid anxiety disorder.^200^ In adolescents, in the tract connecting the sgACC to amygdala in the right hemisphere, FA was lower in MDD compared with healthy controls (HC).^201^ In this study, there was high comorbidity of MDD with ADs, PTSD, attention deficit hyperactivity disorder and past substance abuse. In studies of medication-free adolescents with MDD without significant comorbidities, compared with HC, FA was lower and RD was higher in the bilateral uncinate fasciculi.^202^ In the cingulum, in MDD compared with HC, there was either no difference197 or lower FA,^202,203^ with lower FA correlating with higher MDD severity.^203^

#### Findings in adult patients

Concerning volumetric studies in adult patients with MDD versus HC, there are reports of lower volumes for left pgACC,^204^ bilateral sgACC,^205^ and whole ACC.^206^ For amygdala, a higher bilateral volume was observed in MDD versus HC.^204,205^ Furthermore, these changes co-occurred, with an inverse association between amygdala volume and volumes of dACC and prefrontal cortex including sgACC.^205^ In an MRI study that assessed myelin in white and grey matter using R1 (1/T1), which quantifies longitudinal relaxation rate of water hydrogen protons in a magnetic field, there was no difference in sgACC R1 between MDD and HC when whole-brain R1 was used as a covariate.^207^ In diffusion studies of sgACC-MTL tracts, each of lower FA and higher RD in the right uncinate fasciculus and lower average FA in right and left uncinate fasciculi were observed in MDD compared with HC, indicative of reduced integrity of white matter tracts in MDD.^78,193,208^

In a multimodal imaging study, FA was lower in the second part of the bilateral uncinate fasciculi in MDD versus HC. This co-occurred with higher bilateral sgACC-amygdala FC in MDD versus HC, with a positive correlation between depression severity and sgACC-right amygdala FC.^78^ In this and another study, there was no correlation between uncinate fasciculus FA and MDD severity, although the normalized number of fibres did correlate negatively with MDD severity.^78,193^ In a separate study, FA was lower in subgenual and amygdaloid fibres of the cingulum bundle in MDD versus HC; there was no MDD versus HC FC difference in the main dorsal cingulum body, suggesting that FA changes are specific in MDD.^208^ In another study, in the entire right ACC, FA tended to be lower as the longevity of MDD increased.^206^ In a multimodal study combining volumetry with task fMRI, in MDD but not in HC, higher left amygdala volume was associated with it having less sustained functional reactivity to negative versus positive words.^209^ Regarding spectroscopic findings, in HC, there was a positive correlation between glutamate levels in PFC and FA in the sgACC-amygdala tract, and this was absent in MDD subjects.^210^

In GAD patients, about half of whom had co-morbid MDD, FA was lower in the bilateral uncinate fasciculi relative to HC; the most pronounced FA reduction was in patients without any comorbidity.^211^ In patients with generalized social anxiety disorder without recent episodes of MDD, FA was lower in the right uncinate fasciculus compared with HC.^212^

In one study of PTSD patients, where T1w/T2w ratio was employed as a marker for myelin content as previously described,^213^ amygdala myelin content correlated positively with PTSD symptoms, including avoidance of thoughts, feelings, conversations, diminished interest in activities, psychological and physiological distress at exposure to cues, and startle.^214^

To summarize, in adolescents with MDD versus HC, there is structural imaging evidence for lower ACC volume and lower FA in the uncinate fasciculus and the tract connecting sgACC-amygdala. In the cingulum, FA is either lower or unchanged in MDD versus HC. In adults with MDD, there is structural imaging evidence for lower ACC volume, lower FA in sgACC-amygdala tract, and lower FA and higher RD of the uncinate fasciculus. For amygdala in adulthood, higher volume was observed in MDD compared with HC. Regarding task functional imaging supported by simultaneous structural imaging findings, increased bilateral sgACC-amygdala FC was observed in MDD versus HC, with higher depression severity associated with higher sgACC-right amygdala FC. Also in MDD, higher left amygdala volume predicted its lower sustained functional reactivity to negative words. In adults with ADs, the evidence is for lower FA in the uncinate fasciculi compared with HC.

## Rodent mPFC<->amygdala myelin-related structure-function changes in models of stress-induced hyper-sensitivity to aversion

A number of rodent studies in which stress led to increased aversion sensitivity in one or more behavioural tests (see section 3.2) have investigated and identified alterations in the volume and/or structure of the cortico-limbic system. These studies include pre-adulthood stressors assessed prior to or in adulthood, and adulthood stressors and assessment (Supplementary Table 2). With respect to pre-adult stressors, when rats underwent maternal separation on each of postnatal days P2-P9 and were studied as adults using MRI, DTI and tractography, there was an increase in the number of white matter fibres connecting mPFC and amygdala (BLA and central nucleus).^215^ Rats that experienced limited bedding conditions from P1 to P10 presented with an increased PrL volume at P18 but not in adulthood, and no volumetric changes in IL or BLA.^180^ Rats were exposed to peripubertal stress in the form of OF, EPM and predator odour between P28 and P42, tested behaviourally, and *ex vivo* MRI was conducted at P135 (adulthood): there was no change in FA or MD in mPFC or amygdala in rats that developed anxiety behaviour (FA was reduced in amygdala and MD was reduced in IL and PrL in rats that developed high levels of aggressive behaviour).^184^ In the stress-sensitive Wistar Kyoto rat strain, males were exposed to 3-day unpredictable stress—including restraint, wet cage and elevated platform, at P27-29 or P44-46. Stress at P27-29 (but not P44-P46) resulted in a higher apparent diffusion coefficient (ADC, index of the magnitude of diffusion within the tissue) in amygdala at P60 compared with controls.^216^ In Wistar-strain male rats exposed to the same stressor, those exposed at P44-P46 (but not those at P27-P29) had a lower ADC in amygdala at P60 versus controls.^216^

With respect to stress during adulthood, in rats that underwent chronic unpredictable mild stress (CUMS) followed by behavioural testing and then *ex vivo* T2-weighted imaging and DTI of the whole brain, the volumes of PrL and amygdala were increased compared with controls; further, higher PrL and amygdala volumes correlated with less time spent on the open arms in the EPM test, i.e. higher anxiety, in CUMS subjects.^217^ Whole-brain structural covariance patterns analysis identified decreased global clustering and no changes in global integration capacity, suggesting that it was primarily local processing/networking that was altered in CUMS rats.^217^ An *in vivo* diffusion MRI study identified higher FA in amygdala following CUMS in adult rats.^218^ The CUMS protocol in adult rats has also been shown to change cortical structure: in the frontal cortex, including mPFC, *in vivo* diffusion MRI and DTI revealed increases in axial kurtosis tensor^218^ and MD and reduced FA.^219^ These effects could reflect alterations in dendritic and fibre architecture, and/or a loss of bundle coherence due to demyelination or reduced fibre density.^219,220^ Equally important, some CUMS studies in adult rats report an absence of cortico-limbic volumetric changes. In one study employing diffusion kurtosis imaging and high-resolution MRI, there was no volumetric change in mPFC or amygdala; kurtosis analysis identified no change in radial diffusion in the PFC, including PrL and IL, and an increase in the amygdala.^221^ Heterogeneity of the region of interest included in the study (especially with respect to cortical areas) and differences in the CUMS protocols (e.g. in the social component) could contribute to these inter-study differences.^221,222^

A stressor in the form of a daily period of immobilization on each of 10 days in adult rats did not affect the volume of the cortico-limbic system.^222^ In Fisher 344 (F344) and Sprague–Dawley (SD) adult male rats, a stressor of 15 days of 30-min daily placement on an unsteady and illuminated platform was used:^223^ In both strains, there was a minor reduction in mPFC volume in stressed versus control, and in F344 specifically there was a substantial increase in amygdala volume; F344 rats also showed a more marked plasma corticosterone stress response than did SD rats.^223^ In the same study, *ex vivo*, high-field diffusion MRI and tract-based spatial analysis identified that, in both strains, stress resulted in an increase in FA and decreases in RD and MD in several WM bundles, including mPFC-related and amygdala WM tracts.^224^ In a mouse study, the effects of CSS (see section 3.2) were studied using diffusion-weighted imaging (DWI) and rs-MRI. The CSS mice demonstrated an increase in cingulum FA without any change in axial or RD, and this co-occurred with an increase in PFC-amygdala rs-FC.^51^ A protocol of 10-day chronic social defeat (CSD, broadly similar to CSS) in mice led to an increase in amygdala volume, specifically in susceptible mice (i.e. CSD mice that showed subsequent passive avoidance of the mouse strain used as the stressor).^225^

Therefore, findings from structural and diffusion MRI studies in rodent models of stress-induced hypersensitivity to aversion are rather inconsistent. While there are several reports of changes in mPFC and amygdala volume and diffusivity, the direction and magnitude of such changes are rather heterogeneous between studies; this might indicate that the form, intensity and chronicity of the stressor are important.

Comparing rodent model findings with the human evidence: whereas in the pre-adult phase, MDD patients show lower ACC volume, and lower (or no changes in) FA in white matter tracts connecting ACC to amygdala, in rodents, pre-adult stress leads to temporarily increased PrL volume, increased mPFC-amygdala FC, and alterations in diffusivity in mPFC and amygdala that are dependent on stress-susceptibility. In adults, one feature common to humans and rodents is found at the volumetric level, with most studies in both human MDD and rodent stress models reporting reduced ACC/mPFC volume and increased amygdala volume. Nevertheless, diffusivity-related findings are contradictory, with MDD and AD patients having mostly lower FA in white matter tracts connecting ACC and amygdala, and rodent stress models having mostly higher FA in analogous tracts/connections. For fMRI, an adult human MDD fMRI study found increased sgACC-amygdala FC, and adult stress in mice led to increased PFC-amygdala rs-FC. When comparing human and rodent studies in this context, it is essential to take account of the differences in the time point at which imaging is conducted. In rodents, most measurements are carried out shortly after the termination of the stress protocol, whereas in humans, they are performed following a sustained period of psychiatric disorder and therefore after additional changes in various biological substrates might well have occurred.

## Evidence for and against oligodendrocyte and myelin pathology in anterior cingulate cortex/medial prefrontal cortex<-> amygdala circuitry in stress-related psychiatric disorders

### Human post-mortem studies

Human post-mortem brain tissue studies complement MRI, particularly structural MRI, findings. Concerning the psychiatric disorders that are the subject of this review, most of the histological and biochemical studies have been conducted with post-mortem brain tissue from MDD patients, often those who have undertaken suicide (Supplementary Table 3).

Several MDD and some PTSD studies have investigated region-specific OL-related gene expression, i.e. expression of genes that are enriched in cells of the OL lineage, in patients compared with controls who were not diagnosed with a psychiatric disorder at the time of death. A number report reduced expression of OL-related transcripts in PFC, including ACC, in MDD versus controls, in combined samples of women and men.^60,226,227^ A meta-analysis of eight microarray datasets found sex-specific OL changes in sgACC in MDD: expression of OL-related transcripts was increased in men and decreased in women in patients versus controls.^228^

At the cellular level, disorder-associated changes in overall glial density, OL density specifically and in morphological features, have been demonstrated; the direction of these changes varies across studies. Using stereological histology, one study detected fewer total glial cells (i.e. OL lineage, astrocytes and microglia) in ACC in MDD or another severe psychiatric illness^229^; another study reported such a decrease in ACC of males with familial MDD but not other forms of MDD.^230^ In three further studies, there was no change in glial density in the pgACC in MDD^231,232^ or in suicide completers with a history of MDD,^230^ versus controls. These same studies identified smaller size of glial cells in cortical layers I–III^231^ or higher density of cells with nuclear expression of Olig1^+^ (OL lineage cell marker) in the white matter adjacent to pgACC (but not in the cortical pgACC), in MDD versus controls.^232^ Higher pgACC glial cell density was also found in suicide completers who had a history of MDD and alcohol abuse.^233^ Further cell type-specific histological studies provide evidence that when lower glial cell density is detected in MDD, it is primarily attributable to the OL lineage, including both precursor and mature cells.^234-237^ One study of child abuse and MDD focused on white matter adjacent to pgACC/BA32: it reports lower density of Olig2^+^ (OL lineage marker) cells, higher density of mature OLs and no change in OPC density, in MDD patients who had experienced child abuse versus MDD patients who had not experienced child abuse and controls.^238^ The authors suggest that reduced Olig2^+^ density with no changes in OPCs in child abused-MDD patients reflects depletion of OL lineage cells that were not mature in terms of expression of conventional markers of mature OLs but already at a more differentiated state than OPCs.^238^ In these same subjects, Sox10^+^/Nogo^+^ and Olig2^+^/APC^+^ cells were also lower, suggest that child abuse resulted in a more mature OL lineage phenotype.^238^ The same group, in a study that did not take child abuse into account, identified less connexin-mediated coupling of astrocytes to OLs and in myelinated areas in deep cortical layers of ACC in MDD suicide completers versus controls, suggesting that OL alterations in MDD are mediated, at least in part, by changes in glial coupling and communication.^227^

In amygdala tissue, the status of OLs has been investigated less than in ACC. The small number of studies conducted indicate that OL-related transcripts are less expressed in amygdala of males in MDD^60,228^ and more expressed in amygdala of females in MDD.^228,239,240^ At the cellular level, stereological histology has identified consistent evidence in males of lower densities of total glia and of OLs specifically in MDD.^235,239^

Taken together, human post-mortem findings are rather inconsistent; overall, they allow for the cautious conclusion that in MDD and other stress-related psychiatric disorders, the disorder is associated with altered OL/myelination status in ACC and amygdala. Certain of these changes might contribute to the structural-functional changes observed, and to the pathophysiology of increased aversion sensitivity that is a major feature of these disorders.

### Rodent ex vivo studies

Rodent studies have been conducted to investigate the effects of chronic stressors on the status of the OL lineage and myelin, either prepubertally or in adulthood, including in mPFC and amygdala. The effects of stress can vary substantially depending on whether it took place during critical developmental windows or in adulthood; accordingly, in the following sections, we summarize the effects of stress on OL and myelin considering pre-adult stress and adult stress separately (Supplementary Table 4).

#### Pre-adult stress

In the case of the stressors, maternal separation, limited availability of nesting material to dam and pups, early weaning and peripubertal stress, studies have focused on mPFC. In general, these studies show that changes in mPFC OLs and myelin are already detectable shortly after stressor termination and, furthermore, that they can persist into adulthood. In BALB/cJ male mice, at 1 day after maternal separation (i.e. P15) mPFC transcript and protein levels of the myelin proteins PLP1, MOG and MAG were increased.^181^ In SD male and female rats, the level of mPFC MBP was decreased by maternal separation both at P21 and in adulthood,^241^ as was mPFC MBP in adulthood after maternal separation plus early weaning.^242^ At the histological level, two studies report that maternal separation leads to initial changes in mature OL density^181,241^ and OL lineage proliferation-maturation rate in mPFC,^181,241^ although the directions of such changes versus controls were different between studies: Teissier *et al.*^181^ reported no changes in OPC density and higher mature OL density at P15, while Yang *et al.*^241^ reported higher OPC density and lower mature OL density at P21. That these effects might be temporary and vary across developmental stages is suggested by the absence of an effect of maternal separation on mature OL density in adulthood.^181^ Yang *et al*. also detected higher proliferative OPCs at P21,^241^ while Teissier *et al*. observed lower OPC proliferation at P15.^181^

CSD in juvenile mice, either conventional (10 days of daily defeat) or intermittent (3 days with defeat and 1 day without), led to a smaller area of MBP^+^ staining in mPFC measured at 1 day or 3 weeks after stressor termination^185,243^; this indicates a rather persistent effect of prepubertal experience of social aversion on mPFC myelin content. By contrast, OL density in mPFC was mostly unaffected, either shortly or 3 weeks after stressor termination.^185,243,244^ However, juvenile CSD in mice led to a general reduction in OL lineage proliferation in mPFC at P50.^244^ Social isolation is a manipulation that begins prior to puberty (juvenile-adolescent stage) and is permanent thereafter. In male mice, social isolation from P21 to P65 led in the mPFC to fewer myelin-related transcripts, lower myelin thickness and reduced mature OL morphological complexity at P65.^132^

To our knowledge, there are two reports in rodents on the effects of chronic early-life stress on amygdala OLs (and none on amygdala myelination). In rats, 3 days of repeated variable stress at P27 to P30 (including elevated platform, inescapable footshock and forced swim) led to fewer proliferative OPCs in amygdala,^245^ while 10-day CSD in 4-week-old mice had no effect on OL lineage density and/or proliferation in BLA.^244^

With respect to acute stressors, which might be of particular relevance to PTSD, exposure of 4-week-old rats to acute immobilization stress and predator odour led to effects on the OL lineage and myelin that were sex-, brain region- and time point-specific. Thus, in females at P40, fewer glutathione s-transferase-π (GST-π)^+^ cells, a marker for immature-to-mature OLs, were detected in mPFC but not in BLA, and there was no effect on MBP content.^243^ At P95, less MBP content was observed in mPFC and BLA, and the density of GST-π^+^ cells was unaffected.^246^ In males at P40, more MBP content was detected in BLA with no change in mPFC, and no changes in GST-π^+^ cells were observed.^246^ By contrast, at P95, there was no longer an effect of the stressor on amygdala MBP, and GST-π^+^ cells density was now lower in amygdala.^246^

#### Adulthood stress

In adult rodents, OLs and myelin have been shown to be variously affected by chronic stressors. Molecular studies have identified expression changes in OL- and myelin-related transcripts and proteins in mPFC. Thus, in mice, social isolation or CSS/CSD resident-intruder protocols led consistently to reduced expression of OL-, myelin- and myelin-axon unit-related transcripts.^146,247-249^ In male Swiss Webster mice, 5-week CUMS led to increased *Gpm6a*, *Mal* and *Mog* transcripts in brains collected 2 days later, but only in the substrain specifically bred for low swim stress-induced analgesia and not in the opposite substrain.^250^ Adult male rats exposed to 5-week CUMS had reduced protein levels of MBP, CNPase and Olig2 in mPFC.^251^ One study of adult male mice found that effects of a stressor on myelin-related transcripts in mPFC depended on when within the time period covered by the chronic stressor the effects were assessed: mice were exposed to the unpredictable stress protocol including tail suspension, foot-shock and restraint on a daily basis for between 1 and 4 weeks. In subjects studied at 1 week, myelin-related transcripts were lower versus controls, at 2 weeks, there was no change and at 3 weeks, levels were lower again. It is important to note that these mice also underwent stress-associated behavioural testing.^252^ At the histological level, in mice exposed to 21-day chronic stress (including social defeat, restraint and forced swimming) and subsequent behavioural testing, in mPFC each of reduced morphological complexity of OPCs, density and proliferation of the OL lineage, and MBP intensity, were observed.^253^ Similarly, 5-week CUMS in adult rats led to lower MBP signal and CNPase^+^ cell density in mPFC, suggesting that chronic aversive experiences might impact on OL survival and, consequently, myelin status.^251,254^ Also, 8-week social isolation in adult mice led to lower myelin thickness.^146^

The CSS/CSD resident-intruder protocols have also yielded heterogeneous results with respect to effects on mPFC OL lineage and myelination. A 15-day CSS protocol in young-adult mice led to lower OL-related transcripts in mPFC, including PrL, IL and corpus callosum.^248^ This was not associated with lower mPFC OL density, and appeared to be due to a specific alteration in the OL transcriptome in extant OLs.^255^ At the histological level, this 15-day CSS led to reduced OPC proliferation and higher myelin content in mPFC.^255^ By contrast, 10-day CSD in mice led to an increase, a decrease, or no change in myelin thickness, depending on mouse strain and post-stress behavioural response (active approach to or passive avoidance of) to the aggressor/resident mouse strain.^247,256,257^ One such study reported shorter myelin internodal length,^253^ more OPCs, and lower mature OL density in the mPFC.^256^ In another such study, where the mPFC OL lineage density was assessed histologically at different protocol days, at Day 4 mPFC OPC density was higher versus control, followed by being lower on subsequent days during and after the 10-day protocol.^234^ In another 10-day CSD study, density, morphological complexity and proliferation of OPCs were lower in mPFC, specifically in those mice that passively avoided the aggressor strain in a subsequent social interaction test.^257^ After 14-day CSD, MBP area, myelin fibre density and length were lower in mPFC, with no change in mature GST-π^+^ OL density.^249^ Concerning evidence for causality between OL lineage changes and aversion stimulus processing, in a transgenic mouse line where NG2 cells can be transiently depleted via diphtheria toxin administration, such a depletion of mPFC OPCs was combined with 1 day of social stress: this replicated some behavioural effects of CSS, including increased conflict anxiety in EPM and OF and reduced social interaction.^234^

In amygdala as for mPFC, studies of the effects of stress on OLs and myelin have yielded rather variable findings. With respect to acute stress of relevance to PTSD, in adult male mice, RNA-sequencing of samples collected 2 h after exposure to an unfamiliar conspecific revealed a shift in expression of genes concerned with energy metabolism which was correlated with changes in OL gene expression.^258^ Microarray analysis of amygdala-derived RNA from mice that underwent predator scent-exposure stress revealed no changes in expression of OL- or myelin-related transcripts.^259^ In SD rats aged P65, exposure to predator scent in the form of fox urine was combined with 3 h of immobilization stress, and this was followed by PALM testing: aversion memory expression measured as freezing behaviour was correlated positively with MBP content of amygdala.^214^

With respect to chronic stress and amygdala, male mice exposed to CUMS had lower expression of myelin-related transcripts in amygdala.^60^ Interestingly, the antidepressant fluoxetine (20 mg/kg/day) or a corticotropin-releasing-factor receptor 1 antagonist (SSR125543, 20 mg/kg/day) reversed these effects to some extent.^60^ Male mice that underwent 15-day CSS had lower amygdala expression of myelin and myelin-axon-unit-related transcripts, while the corresponding proteins were unchanged and there was no change in Olig2^+^ OLs.^248^ The same stressor resulted in lower density of proliferative OPCs and more mature OLs at 1 and 15 days after stressor termination.^255^ In male mice, a 4-week variable stress protocol (including daily tail suspension, footshock and restraint) did not lead to any detectable change in OL-related transcripts in amygdala.^252^

Taken together, there is quite extensive evidence in rodents that the status in mPFC and amygdala of the OL lineage and myelin are altered by acute and chronic stressors at the levels of genes and proteins. However, this evidence is inconsistent across studies. Furthermore, changes in OL lineage- and myelin-related transcripts and proteins do not predict changes in OL lineage density or myelin content, respectively, in a consistent manner. Thus, caution is required when interpreting and drawing conclusions based solely on gene expression. Additionally, the inconsistency between studies suggests that the effect of stress on OLs and myelin is influenced by multiple variables, including type and duration of the stressor, inclusion of further behavioural testing during or after the stress protocol, and certainly on the time point relative to the stressor at which the changes are assessed. For instance, with respect to additional behavioural testing carried out during or after stress protocols, it has been reported consistently that exposure to environmental stimuli and behavioural tasks does indeed impact on OL lineage proliferation and maturation dynamics (e.g. see^134,135,137,145,148,149,251^). Thus, synergistic effects of stressors and test-related oligodendrogenesis could underlie the inconsistencies across studies. Furthermore, with respect to effects of the type of stressor and the protocol used, minor changes could have a major impact on OL lineage and myelin. For instance, CSS and CSD procedures overlap substantially. However, whereas the CSS protocol includes the trimming of the teeth of the aggressor to minimize bite wounds during attacks, CSD does not.^170,260^ Peripheral inflammation induced by bite wounds can lead to microglia-mediated inflammation in the brain,^261^ which in turn could alter OL lineage and myelin status.^9,262,263^ Empirical approaches will have to be employed to test these hypotheses, and those protocols that do not minimize wounding will need to be modified to increase their aetiological validity.

Comparing the evidence from rodent models with that from human studies, it is apparent that there is an overall absence of consistency with respect to changes in OL lineage and myelin in ACC/mPFC and amygdala associated with excessive aversion processing. The most consistent finding is that human stress-related neuropsychiatric disorders and adulthood stressors in rodents are associated with lower levels of OL-related transcripts (and proteins). However, even in this regard, there are human gender differences and rodent effects are stress model-specific. Otherwise, most human histological studies have focused on determining either the density of OL lineage cells or myelin content, while on the study of OL lineage proliferation-maturation dynamics is largely missing. This further increases the complexity of comparing findings between human and rodent models. Generally, in the case of human post-mortem samples, it needs to be noted that between-study histological discrepancies could be related to the variable and often extended interval between death and brain collection. This is, of course, not an issue in rodent studies, Comparisons between human and rodent findings can be compromised by the challenges, addressed above, of identifying analogous brain regions in human and rodent brains. Beyond this, there still appears to be a substantial heterogeneity in findings across rodent studies, even though the methods used do not appear to be too different and practical issues faced by human studies can be excluded. Here, the challenge is to identify those stressors with the greatest aetiological validity.

## The current case for ACC/mPFC-amygdala circuit oligodendrocytes and myelination in excessive aversion processing

This review assesses the extent to which current evidence supports or contradicts the inter-related hypotheses that: (i) Human/primate rACC<->amygdala and rodent mPFC<->BLA bidirectional circuits are major contributors to their respective neural circuitries of adaptive aversion processing; (iii) Structural and functional changes in the human rACC<->amygdala circuit and the rodent mPFC<->BLA circuit are associated with the excessive aversion processing that underlies, respectively, major symptoms of stress-related neuropsychiatric disorders in humans and is induced by chronic stressors in rodents; and (iii) Chronic stress-related/induced changes in human rACC<->amygdala and rodent mPFC<->BLA bidirectional circuits are associated with and potentially mediated by changes in OL lineage cells, OLs and myelination.

1. There is strong evidence that the human/primate rACC<->amygdala and rodent mPFC<->BLA bidirectional circuits are major contributors to the respective neural circuitries of adaptive aversion processing. The human evidence indicates that particularly the rACC (pgACC, sgACC) is in a state of altered activity during aversive stimulus processing: Both subregion-specific stimulation and lesioning result in a constellation of changes in emotional feelings and processing. Subregion-specific fMRI studies demonstrate altered activity during each of aversion conditioning, emotional-cognitive resolution of aversion tasks, and fear extinction learning. Amygdala lesioning results in decreased aversion sensitivity, while in fMRI studies the intact amygdala displays increased activity during aversive stimulus processing. The between-region FC evidence for rACC<->amygdala suggests that mutual inhibition is essential for adaptive, situation-specific emotional processing and decision-making. In rodents, PrL and IL are activated by aversion learning and extinction learning, respectively, and BLA is activated during aversion stimulus processing and learning. Bidirectional activity in BLA<->PrL underlies aversion learning and memory expression, and in BLA<->IL underlies aversion extinction learning. As for human rACC-amygdala, the evidence is for mutual regulation in mPFC<->BLA. Therefore, the human rACC<->amygdala and rodent mPFC<->BLA bidirectional circuits are major contributors to the neural circuitry of adaptive aversion processing. However, the structure-function analogy between these human and rodent circuits is limited, and most notably in the extent to which the structural analogy between human sgACC and rodent IL is not reflected by functional analogy.
2. In MDD patients versus controls, there is structural imaging evidence for each of: lower ACC volume; lower FA and/or higher RD or no change in FA in uncinate fasciculus and the tract connecting sgACC-amygdala; lower FA or no change in FA in cingulum; higher amygdala volume. In ADs, there is evidence for lower FA in uncinate fasciculus. In MDD, there is task-fMRI evidence for higher sgACC-amygdala FC in the absence of an association between FC and FA. Rodent models of stress-induced increased aversion sensitivity also provide evidence for altered mPFC and amygdala structure-function, some of which is in the same direction as and some in the opposite direction to the human evidence: Rodent pre-adult stress leads to temporary states of higher PrL volume and higher mPFC-amygdala FC. Adult stress leads to lower volume of mPFC and higher volume of amygdala. In white matter tracts connecting mPFC and amygdala, FA is primarily higher. Therefore, there is some evidence, albeit inconsistent, from diffusion-based MRI studies that FA is altered in the white matter tracts of human rACC<->amygdala and rodent mPFC<->amygdala, implicating altered integrity of the myelination of neurons in these circuits. The evidence is that myelination is decreased in humans and increased in rodents. For rodent resting-state fMRI data, adult stress leads to higher PFC-amygdala rs-FC, suggesting consistency with the evidence for higher sgACC-amygdala rs-FC in MDD.
3. At the post-mortem cellular-molecular level of the OL lineage, OLs and myelination, the major human evidence for MDD and other stress-related disorders comprises: In PFC including ACC, lower expression of OL-related transcripts; either a decrease, increase or no change in the number of glial cells in ACC, with decreases primarily attributable to the OL lineage including precursor and mature cells; in amygdala, either lower or higher OL-related transcript expression, and lower densities of total glia and OLs specifically. In rodent stress models: in mPFC, lower expression of OL-, myelin- and myelin-axon unit-related transcripts; lower density and proliferation of the OL lineage, lower MBP intensity, lower myelin fibre density and length, and lower OPC proliferation combined with higher myelin content; in amygdala, lower myelin- and myelin-axon unit-related transcripts, no changes in the corresponding proteins, and changes in the densities of protein markers of various cell types of the OL lineage.

In summary, the comparative evidence from humans and rodents is that their respective bidirectional rACC-amygdala and mPFC-BLA neural circuits are integral to adaptive aversion processing. These circuits are rather analogous in structural terms but, and in particular with respect to ACC/mPFC subregions, less so in functional terms. Imaging studies have provided evidence that the structure and function of the human rACC-amygdala circuit is altered in stress-related neuropsychiatric disorders characterized by excessive aversion processing, and that the rodent mPFC-amygdala circuit is altered in models of stress-induced increased aversion processing. Structurally, the human MDD and rodent chronic stress evidence is for less and more myelination in the relevant white matter tracts, respectively. Functionally, the human MDD and rodent chronic stress evidence is for higher resting-state FC between sgACC-amygdala and mPFC-amygdala, respectively. Both within and between human and rodent studies, the current evidence for cellular-molecular changes in OL lineage, OLs and/or myelin within these circuits that are associated with stress-related disorders or chronic stress is discordant. While this might indicate that specific stress-induced changes in OLs and myelination are rather random and not causally involved in the aetio-pathophysiology of MDD, ADs or PTSD, this conclusion would be premature. In addition to providing a timely overview of the status of the field, the current review also serves to identify the future studies most needed to clarify the involvement of OLs and myelination. These include: additional and high-resolution human imaging studies of structure and function in ACC-amygdala circuits in patients with excessive aversion sensitivity versus healthy controls; controlled monkey studies of stress effects on aversion processing and ACC-amygdala circuit structure-function, to overcome the limited analogy between rodent and human circuits; controlled rodent studies of stress effects on aversion processing and mPFC-amygdala circuit structure function, supported by investigation of specific OL lineage or myelination manipulations of the mPFC-amygdala circuit on aversion processing.

## Supplementary Material

fcae140_Supplementary_Data

## Data Availability

Data sharing is not applicable to this article as no new data were created or analysed in this study.
