# Supplementary material for: Pathophysiology in cortico-amygdala circuits and excessive aversion processing: the role of oligodendrocytes and myelination
Source: Brain Commun. 2024 Apr 18;6(3):fcae140. doi: 10.1093/braincomms/fcae140 (PMC11073757; doi:10.1093/braincomms/fcae140)
Supplement: fcae140_Supplementary_Data [file fcae140_supplementary_data.docx]

**Supplementary table 1. Summary of human studies of ACC-Amygdala structural and functional connectivity in stress-related psychiatric disorders**

| **Disorder** | **Sex (F/M)** | **Sample size** | **MRI Method** | **Stage** | **Findings for patient group versus HC** | **Comments** | **Reference** |
| --- | --- | --- | --- | --- | --- | --- | --- |
| MDD | MDD (10 males, 22 females), HC (9 males, 13 females) | MDD = 32 HC = 22 | Volumetry | Adolescent | Entire ACC (right h.): decreased volume |  | MacMaster et al., 2014 ^175^ |
| MDD | MDD: 41/39 HC: 52/35 | MDD = 80 HC = 88 | Volumetry | Adolescent | sgACC: no difference | HAMD score inversely correlated with sgACC volume Smaller sgACC volume predicts increased likelihood of comorbid AD | Jaworska et al., 2016 ^176^ |
| MDD | MDD: 9/3 HC: 8/6 | MDD = 12 HC = 14 | DTI | Adolescent | sgACC -Amygdala connection (right): FA decreased | High comorbidity of MDD with anxiety disorders, PTSD, attention deficit hyperactivity disorder and past substance use | Cullen et al., 2010 ^177^ |
| MDD | MDD: 31/1 HC: 26/16 | MDD = 52 HC = 42 | DTI | Adolescent | Uncinate fasciculi (bilateral): decreased FA, Higher RD Cingulum:   no difference in FA | MDD were medication-free | LeWinn et al., 2014 ^178^ |
| MDD | MDD: 8/9 HC: 10/6 | MDD = 17 HC = 16 | DTI | Adolescent | Cingulum: lower FA | MDD were medication-free, depression severity inversely correlated with FA | Henderson et al., 2013 ^179^ |
| MDD | MDD: 10/11 HC: 20/22 | MDD = 21 HC = 42 | Volumetry, DTI | Adult | Entire ACC volume: decreased | trend of inverse correlation of FA in right whole ACC with total days depressed | Abe et al., 2010 ^182^ |
| MDD | MDD: 29/10 HC: 27/7 | MDD = 19 (with childhood abuse) + 20 (without childhood abuse) HC = 34 | Volumetry | Adult | sgACC (bilateral): decreased volume Amygdala (bilateral): increased volume | Volume of AMY to dACC and prefrontal cortex including sgACC inversely correlated; sgACC reduction especially pronounced in MDD patients with a positive family history | Malykhin et al., 2012 ^181^ |
| MDD | MDD: 29/10 HC: 13/5 | MDD = 39 HC = 18 | Volumetry | Adult | Amygdala (bilateral): increased volume dACC (left): decreased volume  pgACC (left): decreased volume | bilateral AMY volume larger in MDD patients with late vs early onset of disease | Vassilopoulou et al., 2013 ^180^ |
| MDD | MDD: 25/14 HC: 27/13 | MDD = 40 HC = 40 | Quantitative MRI (R1) | Adult | sgACC: no difference in R1 |  | Sacchet & Gotlib, 2017 ^183^ |
| MDD | MDD: 54/49 HC: 36/38 | MDD = 103 HC = 74 | DTI | Adult | subgenual and polar stems of the bilateral uncinate fasciculi: decreased FA subgenual and amygdaloid fibres of the cingulum bundle: lower FA in  main dorsal cingulum body: no difference | No diﬀerence between ﬁrst-onset and chronic depression patients | Bhatia et al., 2018. ^70^ |
| MDD | MDD: 9/12 HC: 8/13 | MDD = 21 HC = 21 | DTI | Adult | Uncinate fasciculus (right): decreased FA and increased RD | Normalized number of fibres correlated negatively with depression severity (HAMD) | Zhang et al., 2012 ^170^ |
| MDD | MDD: 14/4 HC: 16/8 | MDD = 18 HC = 24 | DTI and task MRI | Adult | Uncinate fasciculi (right and left): decreased average FA | No correlation between uncinate fasciculus FA and depression severity (HAMD) | de Kwaasteniet et al., 2013 ^71^ |
| AD | AD: 15/15 HC: 20/10 | AD = 30 HC = 30 | DTI | Adult | Uncinate fasciculus (right): decreased FA |  | Phan et al., 2009 ^188^ |
| GAD | GAD: 30/19 HC: 19/20 | GAD = 49 (21 without comorbid depression) HC = 39 | DTI | Adult | Uncinate fasciculi (bilateral): decreased FA |  | Tromp at al., 2012 ^187^ |
| MDD | MDD: 9/21 HC: 16/10 | MDD = 30 HC = 26 | Spectroscopy | Adult | Correlation between prefrontal glutamate levels and FA in Amygdala-sgACC tract in HC only |  | Nugent at al., 2019 ^186^ |
| MDD | MDD: 14/4 HC: 16/8 | MDD = 18 HC = 24 | DTI and task fMRI | Adults | sgACC- bilateral amygdala (bilateral) functional connectivity increased during facial affect processing paradigm | Positive correlation between depression severity (HAMD) and sgACC functional connectivity with right amygdala | de Kwaasteniet et al., 2013 ^71^ |
| MDD | MDD: 3/4 HC: 4/4 | MDD = 7 HC = 8 | Volumetry and task fMRI | Adults | Negative relationship between sustained functional reactivity to negative versus positive words in AMY (left) and the volume of Amygdala (left) in MDD only | Negative relationship between sustained functional reactivity to negative versus positive words in the left amygdala and the volume of the left amygdala in MDD, but not in HC | Siegle at al., 2006 ^185^ |

Abbreviations: MDD (major depressive disorder), AD (anxiety disorder), GAD (Generalized anxiety disorder), HC (healthy control), Fa (fractional anisotropy), DTI (Diffusion Tensor Imaging), HAMD (Hamilton Rating Scale for Depression).

**Supplementary table 2. Summary of rodent studies of mPFC-Amygdala structural and functional connectivity in models of stress-induced increased aversion processing**

| **Species/Strain** | **Sex** | **Sample Size** | **Stress Protocol** | **(St)age at Stressor** | **(St)age at Test** | **MRI Method** | **Findings for stress versus control** | **Comments** | **Reference** |
| --- | --- | --- | --- | --- | --- | --- | --- | --- | --- |
| Rat, Sprague-Dawley | Male | n=8-9/group | Chronic early life stress (CES): (1) plastic-coated aluminum mesh platform ~2.5 cm above the cage floor, (2) reduced bedding material, and (3) reduced nesting material. | P2-P9 | Wk8 (P56-P63) | *ex-vivo* DTI high resolution | ↑amygdala-PFC connectivity | No behavioural testing prior to MRI | Bolton et al., 2018 ^191^ |
| Rat, Wistar Han | Male | n=12/group | Peripubertal stress (PPS): open field, elevated platform, predator odour | Peripubertal: P28-P42 | Adult: P135 | *ex vivo* DTI | = Amygdala FA ↓IL Diffusivity in aggressive rats = PrL diffusivity | (1) Behavioural testing prior to MRI (2) Screened for social aggression | Walker et al., 2018 ^161^ |
| Rat, Sprague-Dawley | Male | Pre-weaning: n=12/group; Adult: n=6/group. | Limited bedding/nesting material paradigm | P1-10 | P18 (pre-weaning) P74-76 (adulthood) | *in-vivo* manganese-enhanced structural MRI | P18: ↑ PrL volume P74-76: = BLA and mPFC volume | Behavioural testing in adults prior to MRI | Guadagno et al., 2018 ^157^ |
| Rat, Wistar-Kyoto & Wistar | Male | *Wistar-Kyoto:*  ES, n=6; LS, n=7; CON, n=6. *Wistar:*  ES, n=6; LS, n=7; CON, n=9. | Elevated platform, restrain and wet cage | P27-P29: early stress P44-P46: late stress | P58-P60 | *﻿in-vivo* DTI | ↑ADC (lateral) Amygdala of ES-WKY rats vs. CON ↓ADC in (lateral) Amygdala ES-W vs. CON. = FA. | Behavioural testing prior to MRI | Zalsman et al., 2015 ^192^ |
| Mouse, Balb/c | Male | n=12/group | Unpredictable Chronic Mild Stress (UCMS) - 5 weeks | 8-week-old | 14-15 week (Experimental day 44) | *ex-vivo* MRI (T2-weighted) | ↑ volume of corticolimbic circuit, including mPFC and Amygdala. ↑Amygdala connectivity to other brain regions | Behavioural testing prior to MRI | Nikolova et al., 2018^193^ |
| Rat, Long Evans | Male | n=8/group | Unpredictable Chronic Mild Stress (UCMS) - 8 weeks | Adult | UMCS: experimental week 18, week 20 and week 25. CON: experimental week 18 and week 22. | longitudinal *in vivo* diffusion MRI (d-MRI), high-resolution T2-weighted images | ↑ Amygdala FA UCMS at week 20 vs. CON ↑ Kurtosis tensor metric WL in the PFC at week 18. ↑Kurtosis tensor metric WL in Amygdala of UCMS at week 20 | (1) Screening for anhedonic vs. resilient (sucrose consumption < 30% vs. <10%) (2) Only anhedonic vs. control animals were included in the MRI study. | Khan et al., 2018 ^196^ |
| Rat, Sprague-Dawley | Male | n=10/group | Chronic Mild Stress (CMS) - 6 weeks | 3-4-week-old | 9-10 week (experimental week 7) | *in-vivo* DTI | In frontal cortex (including mPFC): ↑mean diffusivity, ↑ axial and radial diffusivity, ↓ FA. | Behavioural testing prior to MRI | Hemanth Kumar et al., 2014 ^195^ |
| Rat, Wistar | Male | n=8/group | Chronic Mild stress (CMS) - 8 weeks | 6-7-week-old |  | *In vivo* diffusion kurtosis Imaging and high-resolution MRI | ↑radial diffusivity in Amygdala. = diffusion properties in the PFC. = volumetry. | (1) Screening for anhedonic vs. resilient (sucrose consumption < 50% vs. >50%) (2) Only anhedonic vs. control animals were included in the MRI study. (3) No behavioural testing prior to MRI, except sucrose preference. | Delgado y Palacios et al., 2014 ^197^ |
| Rat, Wistar | Male | n=8/group | 10-day immobilization stress | Adult | 1 day after stress termination | *ex vivo* ﻿high-resolution structural MRI and diffusion kurtosis imaging (DKI) | In cortico-limbic system: =volumetry, = FA, =parallel, perpendicular, and mean kurtosis | No behavioural testing prior to MRI | Henckens et al., 2015^198^ |
| Mouse, C57BL/6 | Male | n=12-19/group | 10-day chronic social defeat (CSD) | 8-week-old | 4 days after stress termination | *ex-vivo* MRI (T2-weighted) and DTI | Social avoidance scores positively correlate with peak voxel volume of Amygdala. Social avoidance scores correlate with FA in Amygdala. | Behavioural testing prior to MRI | Anacker et al., 2015 ^201^ |
| Rat, Sprague-Dawley & Fisher 344 | Male | n=12-15/strain | 15-day of 30-min daily exposure to inescapable stress (rats were placed on an elevated and unsteady platform) | Adult | 24-h after stress termination | *ex-vivo* MRI (quantitative T2* maps) | ↓ mPFC volume in SD and F344. ↑Amygdala volume in F344, but not in SD. | (1) No behavioural testing prior to MRI (2) Cortisone baseline level and post-stress | Bourgin et al., 2015 ^199^ |
| Rat, Sprague-Dawley & Fisher 344 | Male | n=11-13/strain | 15-day of 30-min daily exposure to inescapable stress (rats were placed on an elevated and unsteady platform) | Adult | 24-h after stress termination | *ex-vivo* dMRI and tract-based spatial statistics | ↑ FA and ↓ radial diffusivity and ↓ mean diffusion in several white matter bundles, including the ones related to amygdala and mPFC. | No behavioural testing prior to MRI | Magalhaes et al., 2017 ^200^ |
| Mouse, C57BL/6 | Male | n=16-17/group (2 cohorts) | 15-day chronic social stress (CSS) | Young adults | 24-h after stress termination | Diffusion weighted imaging (DWI) and resting-state functional MRI | In cingulum: ↑FA and = axial and radial diffusivity. ↑FC in cortical network and ↑ FC between PFC and Amygdala. | No behavioural testing prior to MRI | Grandjean et al., 2016 ^50^ |

Symbols and abbreviations: ↑ increased, ↓ decreased, = unchanged, FA (fractional anisotropy)

**Supplementary table 3. Summary of post mortem human studies of ACC-Amygdala oligodendrocytes and myelin in stress-related psychiatric disorders**

| **Disorder** | **Sex (F/M)** | **Sample size** | **Method** | **Stage** | **Findings for patient groups versus HC** | **Comments** | **Reference** |
| --- | --- | --- | --- | --- | --- | --- | --- |
| MDD | men | MDD = 14(AMY)- 16 (ACC) HC = 14(AMY)- 16 (ACC) | ﻿Human Genome U133Plus-2.0 (54,675 probesets or gene transcript levels) | Adult | Reduction of OL-related transcripts in ACC and Amygdala | cause of death for MDD = 50% suicide | Sibille et al., 2009 ^59^ |
| MDD | men | MDD = 16 HC = 14 | Microarray-based expression of QKI | Adult | Reduction of OL-related transcripts in prefrontal cortex (BA56) | Cause of death for MDD: suicide | Klempan at al., 2009^202^ |
| MDD | MDD: 38/10 HC: 18/5 | MDD = 48 HC = 23 | Immunofluorescence and confocal imaging of OL-specific (Cx32 and Cx47) connexins, RNA sequencing, rtPCT | Adult | Reduction of Cx30 expression localized onto OL cells and myelinated fibers in deep cortical layers of the ACC in male-depressed suicides and associated reduction of OL-specific connexin expression. | Cause of death for MDD: suicide | Tanti et al. 2019 ^203^ |
| MDD | MDD: 6/9 HC: 6/9 | MDD = 15 HC = 15 | Stereology | Adult | Reduction of density of glial cells in ACC of patients with MDD | cause of death for MDD = 7/15 suicide | Cotter et al., 2001^205^ |
| MDD | MDD: 1/3 HC: 1/4 | MDD = 4 HC = 5 | Stereology | Adult | Reduction of the number of total glial cells in ACC of males with familial MDD, but not males with other forms of MDD | all data from left hemisphere | Öngür et al., 1998 ^206^ |
| MDD | MDD: 6/9 HC: 6/9 | MDD = 15 HC = 15 | Stereology | Adult | No difference in glial density in the pgACC | cause of death for MDD = 7/15 suicide | Chana et al., 2003 ^207^ |
| MDD | MDD: 7/2 HC: 8/8 | MDD = 9 HC = 16 | Stereology, Olig1-staining | Adult | No difference in glial density in the pgACC | nuclear Olig1-immunoreactivity increased in pACC-adjacent white matter | Mosebach et al., 2013 ^208^ |
| MDD | men | MDD = 13 HC = 13 | Stereology | Adult | No difference in glial density in pgACC between MDD and HC. Increase in glial cell densities in pgACC for alcohol-dependent depressed suicide completers vs depressed suicide completers without alcohol-dependence. | MDD group: cause of death= suicide, (8 present MDD, 5 lifetime MDD) | Hercher at al., 2009 ^209^ |
| MDD | MDD: 4/8 HC: 2/6 | MDD = 12 HC = 8 | Immunohisotchemistry, anti-NG2 staining (precursors for myelinating oligodendrocytes (OLs)) | Adult | Reduction of NG2 glia density |  | Birey et al., 2015^210^ |
| MDD | men | MDD = 36 (18 with and 18 without child abuse) HC = 18 | Stereology | Adult | In white matter adjacent to pgACC reduction of density in cells expressing the OL lineage marker Olig2+, increased density of mature OLs, and no change in OPC density, in MDD patients who had experienced child abuse compared with MDD patients who had not experienced child abuse and controls. Increase in low Sox10+/Nogo+ and low Olig2+/APC+ cells OL. | Cause of death for MDD= suicide | Tanti et al., 2018 ^214^ |
| MDD | MDD: 3/5 HC: 1/9 | MDD = 8 HC = 10 | Stereology | Adult | Reduction of total glia and oligodendrocytes density in Amygdala |  | Hamidi et al., 2004 ^211^ |
| MDD | MDD: 6/9 HC: 6/9 | MDD = 15 HC = 15 | Stereology | Adult | Reduction of numerical density of oligodendroglial cells was found in layer VI of prefrontal cortex |  | Uranova et al., 2004^213^ |
| MDD | MDD: 24/26 HC: 24/26 | MDD = 50 HC = 50 | Microarray-based gene expression | Adult | Increases in oligodendrocyt-related genes in men with MDD and reduction in women with MDD in Amygdala |  | Seney at al., 2018 ^204^ |
| MDD | women | MDD = 21 HC = 21 | Microarray-based gene expression | Adult | Increase of OL-related transcripts in Amygdala |  | Guilloux et al., 2012 ^216^ |

Abbreviations: MDD (major depressive disorder), HC (healthy control), OL (oligodendrocyte), Olig2 (Oligodendrocyte Transcription Factor 2), Sox10 (SRY-Box Transcription Factor 10)

**Supplementary table 4. Summary of ex vivo rodent studies of mPFC-Amygdala oligodendrocytes and myelin in models of stress-induced increased aversion processing**

| **Species/Strain** | **Sex** | **Sample Size** | **Stress Protocol** | **(St)age at Stressor** | **(St)age at Test** | **Method** | **Findings for stress versus control** | **Comments** | **Reference** |
| --- | --- | --- | --- | --- | --- | --- | --- | --- | --- |
| ﻿Mouse, BALB/cJ | Male | n=12/group | Maternal separation | P2 - P14 | P15 | RNA-sequencing -﻿Illumina HiSeq 2500 and validation with qPCR | In mPFC: ↑Plp1, ↑Mog, ↑Mag | No behavioural testing after stress protocol | Teissier et al., 2020^158^ |
| ﻿Mouse, BALB/cJ | Male | n=12/group | Maternal separation | P2 - P14 | P15 | Immunofluorescence | In mPFC: = OPCs density ↑OL ↓OPCs proliferation | No behavioural testing after stress protocol | Teissier et al., 2020^158^ |
| ﻿Mouse, BALB/cJ | Male | n=12/group | Maternal separation | P2 - P14 | Adult | Immunofluorescence | In mPFC: ↓OPCs density, =OL | No behavioural testing after stress protocol | Teissier et al., 2020^158^ |
| Rat, ﻿Sprague Dawley | Male & Female | n=10-12/group | Maternal separation | P3 - P21 | P21 | Immunofluorescence, Western Blot, Transmission Electron Microscopy | In mPFC: ↓MBP, ↑g-ratio , ↑proliferative OPCs, ↑OPCs density, ↓ newly formed OL | No behavioural testing after stress protocol | Yang et al., 2017^217^ |
| Mouse, ﻿C57Bl/6J and DBA/2J | Male | n=6/group | Maternal separation & early weaning (EW) | P2 - P16 & EW at P17 | P75 | Microarray, RNA-seq, Proteomics, DNA-Methylation screening | In mPFC (including PrL, IL and Cg): ↓myelin-related genes (Enpp2, Mag, Mal, Mbp, Mobp, Mog, Omg, Opalin, and Plp1), ↓Mbp. Not mediated by clear changes in DNA methylation. | No behavioural testing after stress protocol | Bordner et al., 2011^218^ |
| Mouse, Balb/c | Male | n=17-19/group (ex-vivo n=5-6/group) | Intermittent adolescent social defeat | P28-P42 | P43 | Histology | In mPFC ↓Mbp density and = mature OL density | Behavioural testing after stress protocol | Zhang et al., 2016^162^ |
| Mouse, Balb/c | Male | n=17-19/group (ex-vivo n=5-6/group) | Intermittent adolescent social defeat | P28-P42 | P63 | Histology | In mPFC ↓Mbp density and = mature OL density | Behavioural testing after stress protocol | Zhang et al., 2016^162^ |
| Mouse, Balb/c | Male | n=4-6/group | Intermittent adolescent chronic social defeat | P28-P42 | P63 | Histology, Western blot | In mPFC ↓Mbp density and = OPCs and mature OL density | No behavioural testing after stress protocol | Xu et al., 2020^219^ |
| Mouse, ﻿C57BL/6JNifdc | Male | n=12/group | 10-day chronic social defeat | P28 | P52 | Histology | In mPFC ↓proliferative OPCs density and = OPCs and mature OL density. In Amygdala there is no effect on proliferation and density of the OL lineage | Behavioural testing after stress protocol | Chen et al., 2023^220^ |
| Mouse, ﻿C57Bl/6 WT or GMO (plp-eGFP) | Male | n=12-16/group (ex-vivo n=3-8/group) | Social isolation | P21 | P65 | qPCR, immunofluorescence, and transmission electron microscopy | In mPFC: ↓myelin-related transcripts, ↓myelin thickness, ↓morphological complexity of mature OL | Behavioural testing after stress protocol | ﻿Makinodan et al., 2012^117^ |
| Rat, ﻿Sprague Dawley | Male | n=16/group (ex-vivo n=8/group) | Repeated variable stress (elevated platform, inescapable shock, forced swim) | P27-P29 | P32 | Immunofluorescence | In Amygdala: no effect on OL lineage proliferation | No behavioural testing after stress protocol | Saul et al., 2015^221^ |
| Rat, ﻿Sprague Dawley | Male | n=16/group (ex-vivo n=8/group) | Repeated variable stress (elevated platform, inescapable shock, forced swim) | P27-P29 | P41 | Immunofluorescence | In Amygdala: ↓proliferative OPCs | No behavioural testing after stress protocol | Saul et al., 2015^221^ |
| Rat, ﻿Sprague Dawley | Male & Female | n=16/group | Acute immobilisation and predator odour | P28 | P40 | Immunofluorescence | in mPFC: = Mbp and ↓OL density in females. In Amygdala: ↑Mbp in males and = mature OL density | No behavioural testing after stress protocol | Breton et al., 2021^222^ |
| Rat, ﻿Sprague Dawley | Male & Female | n=16/group | Acute immobilisation and predator odour | P28 | P95 | Immunofluorescence | in mPFC: ↓ Mbp in females and = mature OL density. In Amygdala: ↓ Mbp in females and ↓ mature OL density in males | No behavioural testing after stress protocol | Breton et al., 2021^222^ |
|  |  |  |  |  |  |  |  |  |  |
| Mouse, ﻿C57BL/6 | Male | n=6/group | 14-day chronic social defeat | 10/12-week-old | 24 h post-defeat | Microarray, fluorescent in situ hybridization, Black Gold-staining, immunofluorescence | In mPFC: ↓ myelin-related genes, ↓myelinated fibre density/length, ↓ Mbp staining area, = mature OL density | Behavioural testing before sample collection | Lehmann et al. 2017^225^ |
| Mouse, ﻿DBA/2NCrl, 129S2/SvPasCrl, BALB/ cAnNCrl, and C57BL/6NCrl; | Male | n=6/group | 10-day chronic social defeat | 6/7-week-old | 24 h post-defeat | RNA-sequencing, qPCR, histology, transmission electron microscopy | In mPFC: ↓ myelin-related genes (in C57Bl6, RNA-seq, correlated to qPCR data), ↑myelin thickness of small caliber axons (in C57Bl6) | Behavioural testing after stress protocol | Laine et al. 2018^223^ |
| Mouse, ﻿C57BL/6 | Male | n=12/group | 14-day chronic social stress | 12/13-week-old | 24 h post-stress | RNA-sequencing, western blotting, histology | In mPFC: ↓ myelin-related genes. In Amygdala: ↓ myelin-related genes; = Mbp, Plp and Cnp protein level. In Amygdala: = OL lineage density | No behavioural testing after stress protocol | Cathomas et al., 2019^224^ |
| Mouse, Swiss Webster | Male | n=15/group | 5-week chronic mild stress | 12-week-old | 48h post-stress | Microarray, qPCR | In mPFC: ↑myelin-related transcripts (*Gpm6a*, *Mal*, *Mog*) only in low swim stress-induced analgesia | No behavioural testing after stress protocol | Lisowski et al., 2013^226^ |
| Rat, ﻿Sprague Dawley | Male | n=17-23/group (ex-vivo n=5-8/group) | 5-week chronic unpredictable stress | 13/14-week-old | 19/20-week-old | Histology, Western blot | In mPFC: ↓ Mbp staining intensity, ↓ Mbp protein level, ↓mature OL, = OPCs | Behavioural testing before sample collection | Luo et al., 2019^227^ |
| Mouse, ﻿C57BL/6 | Male | n=10-12/group (ex-vivo n=5-6/group) | 4-week chronic variable stress | 7/8-week-old | 1-week, 2-week, 3-week post-stress | qPCR | In mPFC: ↓ myelin- and oligodendrocyte-related transcripts 1-week and 3-week post-stress, but not in between. In Amygdala, = myelin- and oligodendrocyte-related transcripts | Behavioural testing before sample collection | Liu et al., 2017^228^ |
| Mouse, ﻿C57BL/6 | Male | n=12/group (ex-vivo n=4/group) | 21-day chronic stress (social defeat, forced swim, restraint) | 8/10-week-old | Post-stress | Histology | In mPFC: ↓OPCs morphological complexity, ↓OL lineage density, ↓proliferative OL lineage, ↓mature OL, ↓Mbp staining intensity | Behavioural testing before sample collection | Yang et al., 2016^229^ |
| Rat, ﻿Sprague Dawley | Male | n=6/group | 35-day chronic unpredictable stress | adult | Post-stress | Histology | In mPFC: ↓Mbp staining density | Behavioural testing before sample collection | Miguel-Hidalgo et al., 2018^230^ |
| Mouse, ﻿C57BL/6 | Male | n=12/group (ex-vivo n=6/group) | 14-day chronic social stress | 12/13-week-old | 1 day and 15-day post-stress | Histology, transmission electron microscopy | in mPFC: ↓proliferative OPCs, ↑myelin content, = OPCs and mature OL density. In Amygdala: ↓proliferative OPCs, ↑mature OL density, = myelin content | No behavioural testing after stress protocol | Poggi et al., 2022^231^ |
| Mouse, ﻿C57BL/6 | Male | n=52-72/group (ex-vivo n=2-11/group) | 10-day chronic social defeat | 8-week-old | Post-stress | Histology, transmission electron microscopy | in mPFC: ↓internodal length, ↓myelin thickness, ↑ OPCs density, ↓mature OL density, exclusively in susceptible mice. | Behavioural testing after stress protocol | Bonnefil et al., 2019^232^ |
| Mouse, ﻿C57BL/6 | Male | n=4/group | 10-day chronic social defeat | adult | During stress and 8- and 10-days post-stress | Histology | in mPFC: ↑ OPCs density after 4-day stress and ↓OPCs density afterwards and post-stress. | Behavioural testing after stress protocol | Birey et al., 2015^210^ |
| Mouse, C57BL/6J (wt), CSPG4- EGFP, FVB.Cg-Tg(Cspg4-EGFP*) HDbe/J), PDGFRα-CreERT2 (Tg(Pdgfra-cre/ERT2)1Wdr. | Male | n=50/group (ex-vivo n=3-5/group | 10-day chronic social defeat | 2/3-month-old | >72h post-stress | Histology | In mPFC: ↓OPCs density, ↓OPCs morphological complexity, ↓proliferative OPCs in susceptible mice. ↑pre-OL and newly formed OL (post-proliferation) | Behavioural testing after stress protocol | Kokkosis et al., 2019 ^233^ |
| Mouse, ﻿C57BL/6 | Male | n=15/group (ex-vivo n=5/time point) | Exposure to unfamiliar conspecific | 9-week-old | 30 min, 50 min and 120 min post-stress | RNA-sequencing | In mPFC: alteration in OL-related transcripts | No behavioural testing after stress protocol | Saul et al., 2017^234^ |
| Rat, ﻿Sprague Dawley | Male | n=20/group (ex-vivo n=10-12/group) | ﻿Exposure to predator scent & 3 h-immobilization | P72 | P85 | Histology | In Amygdala: Mbp intensity correlated with freezing behaviour in fear conditioning, extinction phase. | Behavioural testing after stress protocol | Long et al., 2021^190^ |
| Rat, ﻿Sprague Dawley | Male & Female | n=5/group/sex | Predator scent | Adult | >7days post stress | Microarray | In Amygdala: no effect on myelin- and oligodendrocyte-related transcripts | Behavioural testing after stress protocol | Kim et al., 2020^235^ |
| Mouse, Balb/c | Male | n=18-19/group (ex-vivo n=6/group) | 7-week unpredictable chronic mild stress | Adult | Post-stress | Microarray | In Amygdala: ↓OL- and myelin-related transcripts | Behavioural testing after stress protocol | Sibille et al. 2009^59^ |
| Mouse, C57Bl/6J | Male & Female | n=4-12/group | 8-week social Isolation | Adult | Post-stress | qPCR, western blotting, transmission electron microscopy | In mPFC: ↓OL- and myelin-related transcripts and proteins, ↓myelin thickness | Behavioural testing after isolation protocol | Liu et al., 2012^130^ |

Symbols and abbreviations: Plp1 (Proteolipid protein1), Mbp (myelin basic protein), Mag (Myelin-associated glycoprotein), Mog (Myelin oligodendrocyte glycoprotein), Enpp2 (Ectonucleotide Pyrophosphatase/Phosphodiesterase 2), Mal (Myelin and lymphocyte protein), Mobp (Myelin Associated Oligodendrocyte Basic Protein), Omg (Oligodendrocyte Myelin Glycoprotein), pre-OL (immature oligodendrocytes), OL (oligodendrocyte), OPC (oligodendrocyte precursor cells), ↑ increased, ↓ decreased, = unchanged.
